# 5-Formyltetrahydrofolate promotes conformational remodeling in a methylenetetrahydrofolate reductase active site and inhibits its activity

**DOI:** 10.1016/j.jbc.2022.102855

**Published:** 2022-12-31

**Authors:** Kazuhiro Yamada, Johnny Mendoza, Markos Koutmos

**Affiliations:** 1 Department of Chemistry, University of Michigan, Ann Arbor, Michigan, USA; 2 Program in Biophysics, University of Michigan, Ann Arbor, Michigan, USA; 3 Program in Chemical Biology, University of Michigan, Ann Arbor, Michigan, USA

**Keywords:** structural enzymology, flavin adenine dinucleotide, flavoprotein, folate, ligand-binding protein, methylenetetrahydrofolate reductase, crystal structure, conformational change, enzyme inhibitor, DHFR, dihydrofolate reductase, eMTHFR, *Escherichia coli* MTHFR, FAD, flavin adenine nucleotide, MTHFR, Methylenetetrahydrofolate reductase, PABA, *p*-aminobenzoic acid, tMTHFR, *Thermus thermophilus* MTHFR, TYMS, thymidylate synthase

## Abstract

The flavoprotein methylenetetrahydrofolate reductase (MTHFR) catalyzes the reduction of N5, N10-methylenetetrahydrofolate (CH_2_-H_4_folate) to N5-methyltetrahydrofolate (CH_3_-H_4_folate), committing a methyl group from the folate cycle to the methionine one. This committed step is the sum of multiple ping-pong electron transfers involving multiple substrates, intermediates, and products all sharing the same active site. Insight into folate substrate binding is needed to better understand this multifunctional active site. Here, we performed activity assays with *Thermus thermophilus* MTHFR (tMTHFR), which showed pH-dependent inhibition by the substrate analog, N5-formyltetrahydrofolate (CHO-H_4_folate). Our crystal structure of a tMTHFR•CHO-H_4_folate complex revealed a unique folate-binding mode; tMTHFR subtly rearranges its active site to form a distinct folate-binding environment. Formation of a novel binding pocket for the CHO-H_4_folate *p*-aminobenzoic acid moiety directly affects how bent the folate ligand is and its accommodation in the active site. Comparative analysis of the available active (FAD- and folate-bound) MTHFR complex structures reveals that CHO-H_4_folate is accommodated in the active site in a conformation that would not support hydride transfer, but rather in a conformation that potentially reports on a different step in the reaction mechanism after this committed step, such as CH_2_-H_4_folate ring-opening. This active site remodeling provides insights into the functional relevance of the differential folate-binding modes and their potential roles in the catalytic cycle. The conformational flexibility displayed by tMTHFR demonstrates how a shared active site can use a few amino acid residues in lieu of extra domains to accommodate chemically distinct moieties and functionalities.

One carbon metabolism is a universal process that underpins most of life’s biochemistry and is central to essential and diverse cellular roles including the biosynthesis of the building blocks of life such as DNA, the generation and catabolism of amino acids, and has a master regulatory role *via* epigenetic modifications such as nucleic acid methylation. Indeed, there has been a rash of interest in enzymes critical to one carbon metabolism, given that their disfunction has been implicated in diseased states (1, 2) ranging from cancers (3) to psychiatric illnesses (4). In humans, the folate and methionine cycles are (the) two key pathways in one carbon metabolism; here, one enzyme, methylenetetrahydrofolate reductase (MTHFR), bridges each cycle, allowing one-carbon units (such as methyl-, methylene-, and formyl-groups) carried by folate to be utilized to synthesize central to life metabolic feedstock compounds such as purine bases, thymidylate, and methionine. MTHFR catalyzes the reduction of N5, N10-methylenetetrahydrofolate (CH_2_-H_4_folate) to produce N5-methyltetrahydrofolate (CH_3_-H_4_folate) (5) (Fig. S1). MTHFR requires flavin adenine nucleotide (FAD) as a noncovalently bound cofactor, which acts as an intermediary electron shuttle (Fig. 1*A*). The overall MTHFR reaction follows a ping-pong bi-bi mechanism (6). In the committed reductive half reaction (Fig. 1*A*, top), MTHFR uses NAD(P)H (7, 8, 9) to reduce FAD, and in the oxidative half reaction (Fig. 1*A*, bottom), the reduced FAD transfers a hydride to CH_2_-H_4_folate to produce CH_3_-H_4_folate. Menadione acts as an electron acceptor from the reduced FAD in MTHFR and is thus routinely used as an artificial oxidant in *in vitro* assays to decouple and separately examine both the NAD(P)H-(Fig. 1*A*, top) and CH_3_-H_4_folate–dependent (Fig. 1*A*, bottom) FAD reducing half reactions (5). All MTHFRs share a highly conserved catalytic domain (Fig. S2), despite varying oligomerization states, such as a dimer in *Thermus thermophilus* HB8 (tMTHFR) (8) *versus* a tetramer in *Escherichia coli* (eMTHFR) (7). Eukaryotic MTHFRs contain an additional regulatory domain in which *S*-adenosylmethionine binding induces conformational changes that impact MTHFR catalytic activity (10, 11). This *S*-adenosylmethionine–dependent allosteric inhibition represents a negative feedback loop that regulates one carbon metabolism and methionine biosynthesis (10, 11, 12). MTHFR is metabolically essential to organisms across all domains of life and its conserved catalytic mechanism is still not fully delineated. In humans, there are several patient mutations in said catalytic domain, and bacterial MTHFRs have proven to be a useful surrogate model for understanding their role. Further structural and biochemical characterization is required to address how the enzyme differentially binds and activates its multiple substrates and their intermediates in the active site during the catalytic cycle. These studies are fundamental in allowing to establish how MTHFR activity is regulated in response to the net flux of each part of the methyl cycle it bridges.

**Figure 1:**
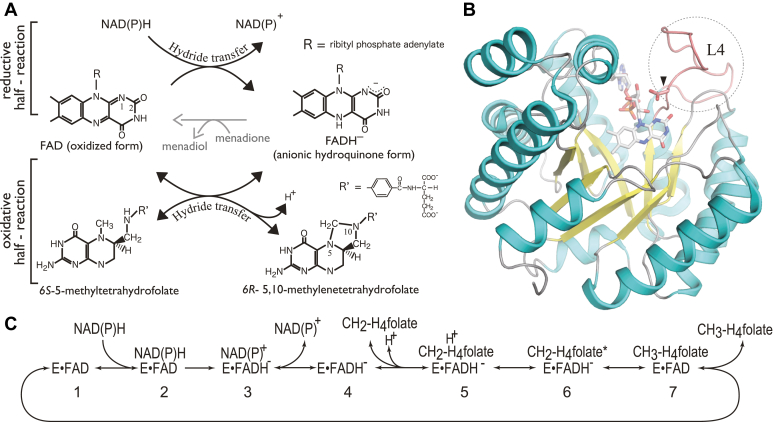
**MTHFR catalyzed half reactions.***A*, in the reductive half reaction, the noncovalently bound FAD is reduced by NAD(P)H. Reduced FAD then reduces CH_2_-H_4_folate to CH_3_-H_4_folate in the oxidative half reaction. Although the folate-dependent half reaction is essentially reversible in bacterial MTHFRs, the NAD(P)H-dependent FAD reduction is irreversible, resulting in an overall reaction that only proceeds in the forward direction under physiological conditions. Menadione is used as an artificial oxidant in two *in vitro* assays; the NAD(P)H:menadione and the CH_3_-H_4_folate:menadione oxidoreductase activity assays. *B*, structure of tMTHFR complexed with FAD (PDB 3APY). Loop L4 is highlighted in *pink*. The arrowhead indicates the invariant and catalytically essential Asp residue (Asp109 and Asp120 in tMTHFR and eMTHFR, respectively). *C*, MTHFR-catalyzed reaction outlining the minimum expected steps and intermediates during catalysis. The NAD(P)H substrate first binds to MTHFR loaded with oxidized FAD (complex 1) to form the enzyme•substrate ternary complex (complex 2), then FAD is reduced to the enzyme•product ternary complex (complex 3). After NAD(P)^+^ release, MTHFR with reduced FAD (binary complex 4) binds CH_2_-H_4_folate to form the second ternary substrate complex (complex 5). A proposed 5-iminium cation CH_2_-H_4_folate intermediate ternary complex forms (complex 6) prior to hydride transfer between folate and FAD. FAD reduces this putative 5-iminium intermediate to form CH_3_-H_4_folate, yielding the final ternary product complex (complex 7). E = enzyme (MTHFR), CH_2_-H_4_folate∗ = putative 5-iminium cation CH_2_-H_4_folate intermediate. FAD, flavin adenine nucleotide; MTHFR, methylenetetrahydrofolate reductase; tMTHFR, Thermus thermophilus MTHFR.

Crystal structures of MTHFR have been previously determined from various species, including *E. coli* (12), *T. thermophilus* (8), *Haemophilus influenzae* (PDB 5UME), human (11), and *Saccharomyces cerevisiae* (11). All MTHFR catalytic domains share the same *β*8*α*8 barrel topology, a typical triose phosphate isomerase barrel structure (13). Structures of the ternary eMTHFR have shown how the enzyme accommodates the NADH and CH_3_-H_4_folate substrates in an overlapping binding site near the *si*-face of the FAD isoalloxazine ring. The protein environment around the ligand-binding site is proposed to support specificity and recognition of the various substrates/products along with their associated intermediates, with the capacity to accommodate, facilitate, and enable multiple charged species transfers (H/e-). In the catalytic cycle, the shared substrate pocket should differentially recognize all substrates such as NAD(P)H, CH_3_-H_4_folate, and CH_2_-H_4_folate, along with their intermediates. The minimum number of unique structures that can accommodate the multiple MTHFR reaction substrates and intermediates and support the multiple catalytic steps are shown in Figure 1*C*. In the folate-dependent oxidative half reaction, there are at least three distinct folate species that are expected to be recognized and differentially activated in the MTHFR active site (Fig. S1) (5). In the first step, the five-membered CH_2_-H_4_folate imidazolidine ring opens by breaking its N5-N10 methylene bridge. CH_2_-H_4_folate ring-opening is facilitated through a preceeding or concurrent N10 protonation and results in the formation of a putative 5-iminium cation folate intermediate (Figs. 1*C* and S1). Subsequent hydride transfer from the reduced FAD (to the 5-iminium intermediate) produces CH_3_-H_4_folate. Because the five-member imidazolidine ring imposes torsional restriction in CH_2_-H_4_folate, a factor not present in CH_3_-H_4_folate, we expect the CH_2_-H_4_folate and CH_3_-H_4_folate–binding modes to be different (14). Similarly, we anticipate that the putative and reactive 5-iminium cation folate intermediate will adopt a different conformation than the other folate substrates, uniquely interacting with its protein surroundings in the active site. Conceivably, local conformational changes are employed to facilitate the transition from a protein imidazolidine ring-opening mode to a hydride transfer mode in the oxidative half reaction without releasing the folate substrate and intermediates. So far, however, the only available structure with folate present is that of eMTHFR complexed with FAD and CH_3_-H_4_folate and the CH_2_-H_4_folate substrate-binding mode of MTHFR (step 5 in Fig. 1*C*) and any other folate-bound intermediate MTHFR structures (summarized as step 6 in Fig. 1*C*) have yet to be determined.

Here, we report the structure of a tMTHFR holodimer in complex with an N5-formyltetrahydrofolate (CHO-H_4_folate) substrate analog. CHO-H_4_folate has a formyl group on the N5 position of tetrahydrofolate (H_4_folate) and was selected for study because it has found use as a nonreactive proxy for CH_2-_H_4_folate (13, 15). Indeed, we find that it exhibits a potent pH-dependent inhibition of tMTHFR activity. We also find that the pterin ring of CHO-H_4_folate stacks against the isoalloxazine ring of FAD, similar to CH_3_-H_4_folate in the eMTHFR structure (14). However, CHO-H_4_folate is found in a more bent shape than CH_3_-H_4_folate. The active site appears to remodel around the PABA folate moiety when CHO-H_4_folate is present, as evidenced by the repositioning of multiple invariant residues. We describe additional subtle structural changes in the folate and FAD vicinity that affect their relative positioning and include the engagement of an invariant Thr with a catalytically crucial Glu in the active site Ser-His-Glu triad. These small structural changes in the active site appear to ultimately reshape the electrostatic (micro)environment near the folate ligand. Together, our findings suggest that CHO-H_4_folate induces active site rearrangements as a result of folate-binding, resulting in a unique MTHFR folate-binding mode. We describe the possible functional significance of the CHO-H_4_folate-binding mode found in tMTHFR and the potential mechanistic role of an observed active site Ser-His-Glu-Thr quartet. MTHFR employs a “Spartan strategy” (14) to create suitable pockets for the chemically different substrates, using limited numbers of amino acid residues rather than additional domain rearrangements. Our work expands on the conformational flexibility displayed by tMTHFR and demonstrates how the shared active site uses the differential positioning of select amino acid residues to accommodate chemically distinct moieties and functionalities.

## Results

### CHO-H_4_folate inhibits tMTHFR activity

**Figure 2:**
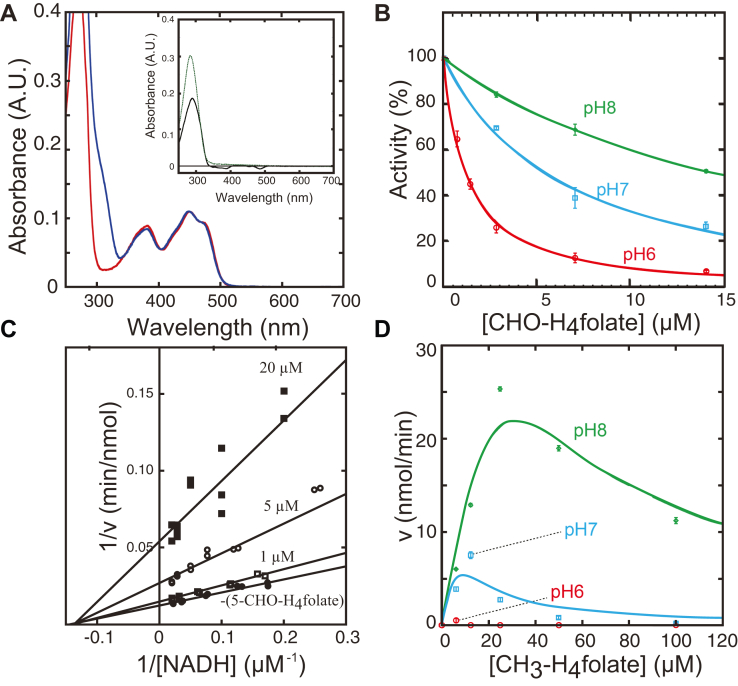
**tMTHFR•CHO-H**_**4**_**folate complex formation and effects of pH and folate on tMTHFR Activity.***A*, UV-VIS spectra of the tMTHFR•CHO-H_4_folate complex. UV-VIS spectra of tMTHFR with (*blue*) or without (*red*) CHO-H_4_folate treatment. The *solid black line* in the inset corresponds the calculated spectrum resulting from subtracting the spectrum of folate-free tMTHFR (*red line*, as a reference) from that of the tMTHFR•CHO-H_4_folate complex (*blue line*). The *dotted green line* represents a free CHO-H_4_folate spectrum. *B*, effect of pH on NADH:menadione oxidoreductase activity. The tMTHFR activity was measured in varied pH units and at room temperature. Equation 1 was used to fit data (43). The vertical axis represents the percentage of the uninhibited initial velocity. Relative activities of tMTHFR at pH 6.0 (*red circles*), 7.0 (*blue squares*), and 8.0 (*green diamonds*) in the presence of varying concentrations of free CHO-H_4_folate are shown (43). The bars represent the SD of triplicate measurements. *C*, double-reciprocal plots of NADH-menadione oxidoreductase activities of tMTHFR in the presence of (*6RS*)-CHO-H_4_folate. Relative activities of tMTHFR in the presence of varying concentrations of free CHO-H_4_folate are shown: at 0 μM (*closed circle*), 1 μM (*open square*), 5 μM (open circle), and 20 μM (*6S*)-CHO-H_4_folate (*closed square*) respectively. *D*, effect of pH on the CH_3_-H_4_folate:menadione oxidoreductase assay. tMTHFR activity, measured by the formation of CH^+^ = H_4_folate as a function of increasing CH_3_-H_4_folate concentration, was performed in triplicate at 50 °C. The bars represent the SD of triplicate measurements. Equation 2 was used to fit data (34). Color scheme is the same as in Panel *B*. MTHFR, methylenetetrahydrofolate reductase; tMTHFR, Thermus thermophilus MTHFR.

In this study, we used CHO-H_4_folate as a folate substrate analog to interrogate tMTHFR function. CHO-H_4_folate calcium salt, also known as Leucovorin calcium, is an FDA-approved compound. It is “widely” used to ameliorate the toxic effects of high-dose folate antagonist chemotherapy by putatively participating in the folate one-carbon pool and bypassing the need for dihydrofolate reductase (DHFR) activation (15). Indeed, several studies interrogating the structure and function of folate-binding enzymes, particularly those that bind CH_2_-H_4_folate (methenyltetrahydrofolate synthase (16), thymidylate synthase (TYMS) (17), DHFR (18, 19), and serine hydroxymethyltransferase (20, 21)) use CHO-H_4_folate as a surrogate, owing to its increased stability relative to other folates in solution and its ability to directly interact with said folate-binding enzymes. However, despite its biological relevance, the effects of CHO-H_4_folate on the biochemical properties of MTHFR have not yet been reported or characterized. We first sought to address whether tMTHFR interacts with CHO-H_4_folate in solution. Figure 2*A* shows the UV-VIS spectra of tMTHFR before and after treatment with CHO-H_4_folate and subsequent gel filtration chromatography to remove free ligand. In addition to the typical UV-VIS absorption corresponding to oxidized flavin (peaks at ≈380 and 450 nm), an additional shoulder peak was evident around 290 nm after treatment with CHO-H_4_folate.

The tight CHO-H_4_folate binding indicates that the folate analog can strongly bind to tMTHFR in solution. Subsequently, we wanted to establish the effect of CHO-H_4_folate binding on tMTHFR activity. Substrate inhibition/competitive substrate inhibition is often observed in enzymes that utilize a ping-pong mechanism. tMTHFR in particular exhibits strong substrate inhibition based on a CH_3_-H_4_folate:menadione oxidoreductase assay, which reports on the reverse MTHFR oxidative half reaction (8). In addition to the known MTHFR inhibition by its substrates (7, 8), other structurally similar substances can bind to the MTHFR active site and inhibit its activity, including various naturally occurring folate species in folate metabolism (6). Therefore, we first sought to address whether CHO-H_4_folate could act as an inhibitor and affect the tMTHFR NADH-dependent reductive half reaction (NADH:menadione oxidoreductase activity) by obstructing tMTHFR•NADH complex formation.

We confirmed that CHO-H_4_folate inhibits activity in tMTHFR based on our NADH:menadione oxidoreductase steady-state assay (Fig. 2*B*). We wanted to determine the mode of inhibition and therefore generated double-reciprocal plots using NADH concentration *versus* velocity curves. Interestingly, the determined double-reciprocal plots of MTHFR activity at different CHO-H_4_folate concentrations indicate noncompetitive inhibition (Fig. 2*C*). This mode of inhibition suggests that CHO-H_4_folate can bind both to the E(nzyme) and the E(nzyme)S(ubstrate) complex, suggesting that in the reductive half reaction, CHO-H_4_folate can compete with NADH for binding to the active site pocket but can also bind to the tMTHFR•NADH complex in a mode that we have yet to structurally characterize. To our knowledge, there is no report describing MTHFR inhibition by CHO-H_4_folate. Based on preliminary experiments, the NADPH:menadione oxidoreductase activity of recombinant human MTHFR (22) is much less sensitive to CHO-H_4_folate, indicating tMTHFR is particularly sensitive to CHO-H_4_folate. In the presence of CHO-H_4_folate, the NADPH:menadione oxidoreductase activity of expressed and purified human MTHFR (22) was lowered to 94% at pH 7.2 based on single point activity measurements. However, in the presence of CHO-H_4_folate at the same pH, tMTHFR NADH:menadione oxidoreductase activity was decreased to 60%.

### Effects of pH and CHO-H_4_folate on tMTHFR activity

We further investigated the effect of pH on the tMTHFR activity inhibition by CHO-H_4_folate since pH can be critical in influencing protein conformations and active site arrangements. Figure 2*B* shows the NADH:menadione oxidoreductase activity of tMTHFR in varying pHs and concentrations of CHO-H_4_folate. At pH 6.0, CHO-H_4_folate inhibits enzyme activity, even at low concentrations (2.8 μM) (Fig. 2*B*, red line). As the pH is increased, the CHO-H_4_folate tMTHFR inhibitory effect is less pronounced (pH 7 and pH 8, blue and green lines, respectively, in Fig. 2*B*). The enzyme activity (the NADH substrate consumption per minute) without CHO-H_4_folate at pH 6.0 was used as a reference point and set as 100%, while the MTHFR activity at pH 7.0 and pH 8.0 was 95% and 120%, respectively. When CHO-H_4_folate was present at 14 μM (the highest concentration in our experimental conditions), the activity at pH 6.0, 7.0, and 8.0 was 9%, 23%, and 60%, respectively, as compared to the activity in the absence of CHO-H_4_folate. This suggests that the effect of pH on the enzyme activity is minimal when CHO-H_4_folate is absent; however, in the presence of CHO-H_4_folate (comparison at the fixed inhibitor concentration, in this instance, 14 μM), the enzyme activity was largely influenced by pH. Our data indicate that solution pH may affect the tMTHFR•CHO-H_4_folate complex stability and therefore impact activity as determined with our NADH:menadione oxidoreductase assay. In the presence of CHO-H_4_folate, at low pH, tMTHFR is trapped in a tMTHFR•CHO-H_4_folate inhibitory complex, which prevents tMTHFR•NADH complex formation because NADH and folate share the same single substrate-binding pocket in MTHFR. As the pH is raised, this tMTHFR•CHO-H_4_folate inhibitory complex can more readily disassemble, allowing for the formation of a productive tMTHFR•NADH complex.

### tMTHFR substrate CH_3_-H_4_folate inhibition is pH-dependent

Given that tMTHFR binds CHO-H_4_folate tightly (Fig. 2*A*) and that it inhibits tMTHFR activity (Fig. 2*B*), we contemplated how it may relate to the previously described role of CH_3_-H_4_folate as a potent substrate-inhibitor in the CH_3_-H_4_folate:menadione oxidoreductase activity assay (8). We propose that MTHFR accommodates folate species in at least two distinct binding modes. We posit that a nonconducive to hydride transfer folate-binding mode is related to the observed substrate inhibition and that said binding mode could be captured in a tMTHFR•CHO-H_4_folate complex. The other distinct binding mode is the CH_3_-H_4_folate–binding pose found in the eMTHFR•CH_3_-H_4_folate structure (productive). We found that tMTHFR•CHO-H_4_folate complex inhibition is pH-dependent, and therefore we hypothesized that the complex formation of tMTHFR•CH_3_-H_4_folate in the CHO-H_4_folate–binding mode is similarly pH sensitive. If so, the substrate inhibition by CH_3_-H_4_folate in the CH_3_-H_4_folate:menadione oxidoreductase assay could likewise be pH-dependent.

In order to examine this hypothesis, CH_3_-H_4_folate:menadione oxidoreductase activity was measured at various pHs (Fig. 2*D* and Table S2). In Figure 2*D*, the *velocity* (the product formation per minute) is plotted *versus* CH_3_-H_4_folate substrate concentrations, and curves of the CH_3_-H_4_folate:menadione oxidoreductase activity in varying pHs are displayed. At pH 6.0, surprisingly, we detected little product formation at concentrations of CH_3_-H_4_folate between 0 and 100 μM. In contrast, as the pH is increased, the inhibitory effect is less pronounced (pH 7 and pH 8, blue and green lines, respectively, in Fig. 2*D*), and substrate inhibition was observed only at high CH_3_-H_4_folate substrate concentrations (100 μM for pH 8). The pH dependence of the substrate inhibition by CH_3_-H_4_folate follows a similar trend to that of NADH:menadione oxidoreductase activity inhibition by CHO-H_4_folate (Fig. 2*B*). It seems that at pH 6.0, almost all tMTHFR•CH_3_-H_4_folate forms an inhibitory or nonproductive complex. However, at higher pHs and especially at pH 8.0, the activity is less inhibited, indicating that tMTHFR is active because the enzyme could form a reactive complex with CH_3_-H_4_folate. The pH dependence of tMTHFR inhibition (Fig. 2, *B* and *D*) by CHO-H_4_folate and CH_3_-H_4_folate lends credence to the notion that tMTHFR exists as a conformational ensemble and exhibits conformation sampling (akin to DHFR (18)), with differing capabilities to bind and catalyze at least three distinct folate-binding conformations; the relative population/conformational dynamism is seemingly pH-dependent. The structural and functional implications of the observed pH-dependent inhibition data are explored in further detail in a subsequent section.

### tMTHFR•CHO-H_4_folate ternary complex structure

Mechanistic studies suggest that the MTHFR active site must be capable of accommodating at least three structurally distinct folate species during the oxidative half reaction (Fig. 1*C*) (5). We hypothesized that active site residue arrangements will be needed to accommodate both binding of the distinct folate species and the reversible transitions between them. The only available MTHFR structure with a folate bound is that of the ternary eMTHFR complex with CH_3_-H_4_folate. Additional structural insights into how other folate species are bound by MTHFR are needed to ultimately understand the MTHFR catalytic cycle (Fig. 1). With this in mind, and given the potent inhibitory effect of CHO-H_4_folate on tMTHFR activity, we obtained the structure of tMTHFR cocrystallized with CHO-H_4_folate.

In the absence of folate, two different FAD-bound tMTHFR structures with different intermonomer interfaces have been described (8). In one of the structures, only one active site in the dimer is occupied by FAD (half-FAD occupancy, dimer mode 1) while both active sites are occupied in the other (full-FAD occupancy, dimer mode 2). It is worth noting that tMTHFR purifies in the half-FAD occupancy state; only the addition of excess FAD leads to the observance of the full-FAD occupancy state. We found that in the presence of CHO-H_4_folate, the tMTHFR dimer adopts the same intermolecular interface as the tMTHFR with half-FAD occupancy (dimer mode 1) (Figs. S3–S5), despite the presence of FAD in both active sites. Compared to the fully loaded FAD-bound tMTHFR structure, CHO-H_4_folate binding did not affect the backbone atom interactions and positions in the inner eight *β*-strands, (r.m.s.d. = 0.479 based on 41 C*α* atoms), suggesting that the *β*-sheet structure of the *β*8*α*8 barrel, where the active site is located, is rigid and remains unperturbed (Fig. S6). However, positions of *α*-helixes in the outer barrel are altered in the presence of CHO-H_4_folate, which could cause a steric clash at the dimer interface, altering the intermolecular interface between dimer mode 2 to dimer mode 1, even though both monomers bind FAD (Figs. S4 and S5). The available tMTHFR structures suggest that the intermolecular dimer interface can adopt two distinct interchangeable conformations; however, the physiological significance of these two conformations is still unclear. The CHO-H_4_folate-induced conformational change to dimer mode 1 was somewhat unexpected since in mode 2, the tMTHFR dimer adopts a more extensive dimer interface mainly due to the presence of an intermolecular β-sheet (β7'A and B in Figs. S3 and S4). The overall dimer structures of tMTHFR with or without folate are illustrated in detail in the Supporting Information (Figs. S3–S5). However, here, we focus on the novel folate-binding mode of CHO-H_4_folate in the active site of tMTHFR.

### MTHFR accommodates multiple folate-binding modes

**Figure 3:**
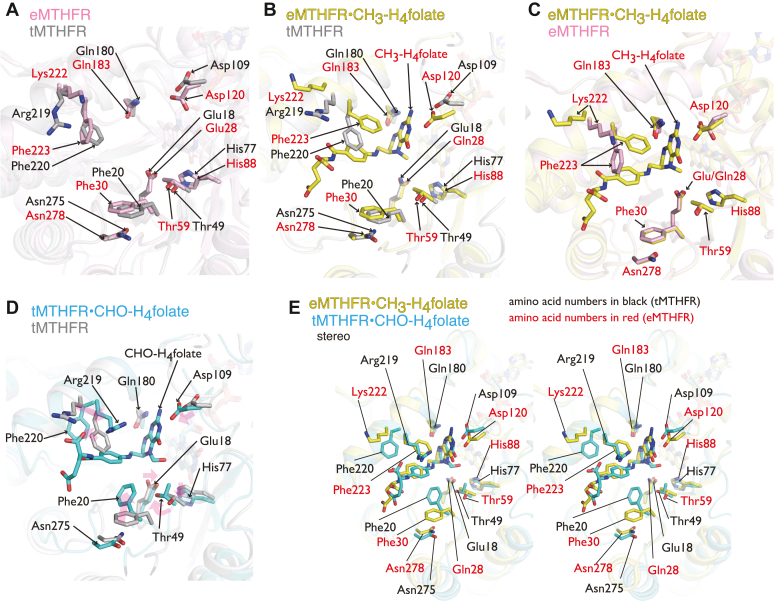
**MTHFR active sites close-up views demonstrating folate-induced active site changes and the differential protein arrangement around the different folate ligands.***A*, structural comparison of tMTHFR (*gray*) and eMTHFR (*pink*) active sites without folate ligands. *B* and *C*, the eMTHFR^Glu28Gln^•CH_3_-H_4_folate (*yellow*) complex structure is superimposed onto folate-free tMTHFR (*gray*) in *B* and onto folate-free eMTHFR (*pink*) in *C*. *D*, conformational changes in tMTHFR upon CHO-H_4_folate binding. Folate-free tMTHFR (*gray*) and the tMTHFR•CHO-H_4_folate complex (*cyan*) are superimposed. *Transparent magenta arrows* indicate conformational changes upon ligand binding. *E*, structural comparison of the different folate-binding modes in eMTHFR^Glu28Gln^•CH_3_-H_4_folate and in tMTHFR•CHO-H4folate represented in a stereo mode. All protein structures are displayed as cartoons with folate ligands and selected active site residues displayed as *sticks*. *Red* and *black* labels correspond to eMTHFR and tMTHFR residues, respectively. MTHFR, methylenetetrahydrofolate reductase; tMTHFR, Thermus thermophilus MTHFR.

MTHFR recognizes and accommodates at least three structurally distinct folate species during the oxidative half reaction (Fig. 1*C*). In the ternary complex of tMTHFR with FAD and CHO-H_4_folate bound (referred to as “tMTHFR•CHO-H_4_folate” in this paper), we observe that the folate pterin ring is stacked against the *si*-face of the FAD isoalloxazine ring. This is similar to the positioning of the CH_3_-H_4_folate pterin ring bound to eMTHFR (14). The active site comparison of the tMTHFR•CHO-H_4_folate and eMTHFR, FAD, and CH_3_-H_4_folate (referred to as “eMTHFR•CH_3_-H_4_folate”) ternary complexes are presented in stereo mode (Fig. 3). The Glu28Gln mutant eMTHFR, whose catalytic activity in the folate-dependent oxidative half reaction is completely abolished, was used to capture and trap the CH_3_-H_4_folate complex. Though previous attempts have been made to obtain a CH_2_-H_4_folate ternary complex with this inactive variant, they proved unsuccessful. The authors (14) noted that this was likely due to the nature of the observed PABA pocket in the oxidized enzyme and posited a distinct PABA pocket was required for proper binding of the substrate. However, this could also indicate that Glu (28 in eMTHFR and 18 in tMTHFR, as shown in Fig. S2) is critical for the folate-dependent reaction in both directions (reversible steps 5–7 in Fig. 1*C*) and therefore involved in either hydride transfer and/or iminium formation/stabilization (14, 23). Considering these previous findings and the successful cocrystallization of tMTHFR with a native substrate analog in CHO-H_4_folate, affording our tMTHFR•CHO-H_4_folate ternary complex, the nature of the PABA pocket was closely assessed.

A close inspection of the active site, as shown in Figure 3, reveals a host of similar and conserved interactions between active site residues and the folate ligands. For example, Asp and Gln residues (Asp109, Gln180 in tMTHFR and Asp120, Gln183 in eMTHFR) provide anchoring bidentate interactions with the pterin ring in both structures. Despite the similarities, there are several distinct differences in the folate-binding modes, with a rather notable one related to the protein pocket in where the folate PABA moiety resides. In the eMTHFR•CH_3_-H_4_folate structure, Phe223 interacts with the PABA ring from the upper side, as viewed in Figure 3, which forms part of the hydrophobic enclosure that it resides in. However, in the tMTHFR•CHO-H_4_folate structure, the upper side of the PABA ring is occupied by Arg219 with Phe220 (corresponding to Phe223 in eMTHFR) relocating but still engaging, albeit from the side rather than from above, with the PABA ring. Moreover, another aromatic residue, Phe20, in tMTHFR•CHO-H_4_folate also rearranges, moves closer, and *pi*-stacks against the PABA moiety from the opposite site of Arg219. As a result, a unique PABA pocket is observed in the tMTHFR•CHO-H_4_folate structure, in where Phe20 and Arg219 now sandwich and enclose the PABA moiety. The unique protein arrangement around PABA and its functional implications are further discussed in a subsequent section.

Upon CH_3_-H_4_folate binding to eMTHFR, Asp120, Lys222, and Phe223 reposition to engage and interact with the CH_3_-H_4_folate substrate (Fig. 3*D*). The local conformational changes in the active site upon binding CHO-H_4_folate in tMTHFR (Fig. 3*E*) are even more striking than in eMTHFR (Fig. 3*D*). In addition to the rearrangement of Asp109, Arg219, and Phe220 (Asp120, Lys222, and Phe223 respectively in eMTHFR), Phe20 and Thr49 are also found in different positions in the tMTHFR•CHO-H_4_folate structure compared to the eMTHFR•CH_3_-H_4_folate one. The repositioning of Thr49 in the tMTHFR•CHO-H_4_folate structure is of particular note. The Thr49 hydroxyl group hydrogen bonds with the Glu18 side chain in the tMTHFR•CHO-H_4_folate structure, a residue whose polarity, as previously mentioned, is essential for catalysis (23) As a result of the Thr49 relocation and its hydrogen bonding with Glu18, an extended quartet is now formed in the active site that also includes His270 and Ser16. Conceivably, the repositioning of Thr49 to engage and perhaps modulate Glu18 electronegativity could relate to and play an important role in regulating MTHFR catalytic function. We further discuss the potential functional significance of Thr49 in a subsequent section. In sum, the comparative analysis of the available MTHFR folate structures reveals differential folate-binding modes as shown in Figure 3.

### Active site remodeling around the folate PABA ring

**Figure 4:**
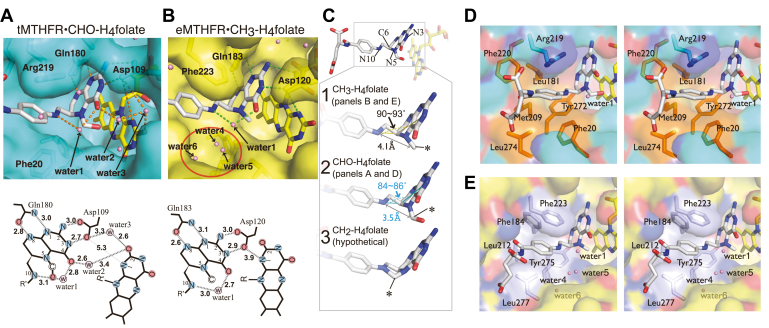
**Differential MTHFR folate-binding modes and water arrangements in the active site proximal to the FAD cofactor.***A* and *B*, folate-binding sites in tMTHFR (*light blue*) and eMTHFR (*yellow*) respectively are shown in the *upper panels*. A schematic representation of the arrangement and interactions/distances between the folate and FAD ligands with nearby waters and loop L4’s Asp, in the active sites of tMTHFR (*left*) and eMTHFR (*right*) is shown in the *lower panels*. *C*, folate arrangements in MTHFR active sites. CH_3_-H_4_folate and CHO-H_4_folate bound in eMTHFR and tMTHFR respectively are illustrated in Panels *C*1 and *C*2. CH_2_-H_4_folate substrate model generated using CHO-H_4_folate in the tMTHFR•CHO-H_4_folate structure as a mold (Panel *C*3). The angle between the PABA moiety and pterin ring is indicated with a *dotted arrow*. The *asterisk* denotes the carbon atom (C11) attached to the folate N5, whereas the *double side arrow* highlights the distance between N10 and C11. *D* and *E*, close-up of MTHFR protein environment around the folate PABA moieties. The hydrophobic residues surrounding PABA are shown as *orange* in *D* for tMTHFR and as *light blue* in *E* for eMTHFR. Protein views are displayed in a semi-transparent surface mode, while amino acids and ligands are displayed as *sticks*. FAD, flavin adenine nucleotide; MTHFR, methylenetetrahydrofolate reductase; PABA, *p*-aminobenzoic acid; tMTHFR, Thermus thermophilus MTHFR.

A protein hydrophobic pocket surrounding the folate PABA ring is observed in folate-dependent enzymes including DHFR (24), TYMS (25), and CH_2_-H_4_folate dehydrogenase/cyclohydrolase (26). In our tMTHFR•CHO-H_4_folate structure, the PABA-binding pocket is composed of Phe20, Met209, Phe220, Arg219, and Leu274 (Fig. 4, *A* and *D*). Comparison of the PABA-binding pockets in the tMTHFR•CHO-H_4_folate and the eMTHFR•CH_3_-H_4_folate reveals that these proteins interact with a different set of invariant active site Phe residues (Figs. 3 and 4). In eMTHFR, Phe223 contacts the PABA ring from the upper side, as seen in Figure 3*A*, while in tMTHFR the interaction with Phe (220 in tMTHFR) is replaced by one with an Arg residue (Arg219). Furthermore, a different Phe residue (Phe20 in tMTHFR, and Phe30 in eMTHFR) approaches and stacks with PABA ring from the reverse side of the Phe223-contacting side in eMTHFR (Figs. 3 and 4).

It is intriguing that Phe20 in tMTHFR•CHO-H_4_folate participates in the PABA pocket formation because this Phe residue faces away from PABA and typically interacts with a nearby Asn residue in previously determined MTHFR structures (with or without substrates). The eMTHFR structure in Figure 3 demonstrates an example of the amide-*pi* interaction between these Phe and Asn residues (Phe30 and Asn278 in eMTHFR). The Phe20 residue is mostly conserved from bacteria to human MTHFRs, with only a few exceptions. The rare example is *Leishmania infantum* MTHFR, in which the Phe residue is replaced but could be functionally conserved by a Tyr residue (Tyr24, NCBI: XP_001469364.1). The Asn residue is strictly conserved in MTHFRs. The patient mutation in this conserved Asn residue (Asn324 in human MTHFR) to Ser causes severe MTHFR-deficiency (27), suggesting that the Asn residue is crucial for proper protein function. Conceivably, the Asn residue orients and “holds” Phe20 away from the PABA moiety and “releases” it when the Phe20-containing PABA pocket is required. The Asn-Phe amide-*pi* interaction is not present in the holo-monomer of the folate-free dimer mode 2 tMTHFR structure (8). Since the Phe-Asn interaction is “disrupted” and has so far been observed only in structures from crystals grown at low pH (4.3∼4.5), the Phe20 release from Asn275 may be pH-dependent (Fig. 3*D*).

As a result of the active site remodeling in the PABA ring vicinity, the environment becomes more hydrophobic and a number of waters in the previously hydrophilic nook right under the PABA ring, as displayed in Figure 4*B*, are excluded with their space now occupied by Phe20 (Fig. 4*B*, highlighted with a red circle). Though the loss of a Phe-Asn amide-*pi* interaction might be slightly deleterious, one could argue that the entropic benefit of a Phe20-lined hydrophobic nook compensates for this loss. The novel PABA pocket also places several aromatic residues near the PABA ring that can *pi*-stack (Phe20 in a tilted manner is ∼3.7 Å away, and Phe220 in a tilted T-shaped manner is ∼3.9 Å away); Arg219 (cation-*pi*) and Met209 (Met-aromatic) provide further stabilization of the PABA moiety. The water rearrangement is of potential significance, since changes in the water environment can influence the flexibility of the loop region (L4) that carries Asp109, which is essential not only for binding but also for activation of folate (14, 28) in the folate-dependent reaction *via* electrostatic stabilization of the putative iminium intermediate. It is worth noting that the L4 loop is found in the “open” position, despite having folate bound. The significance of this change in microenvironment with regards to its effect on folate–FAD interactions is discussed below. In addition, it is of note that the previously mentioned Ser16-His270-Glu18-Thr49 quartet forms near the pterin ring of folate and the isoalloxazine ring of FAD. While the Ser-His-Glu triad interaction is observed in all MTHFR structures, the Glu-Thr interaction in the tMTHFR•CHO-H_4_folate complex is unique.

### Relative orientations of pterin and isoalloxazine rings in the different folate-bound MTHFR structures

**Figure 5:**
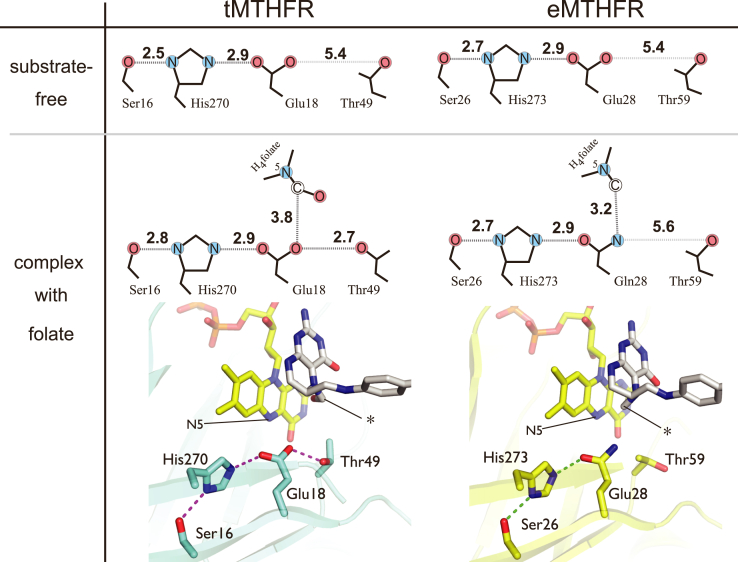
**Quartet formation by Ser-His-Glu and Thr.** Interacting Ser, His, Glu, and Thr residues in eMTHFR (*left*) and tMTHFR (*right*) are shown in schematic mode. The *top row* shows the distances of side chains in the folate-free structures. In the *bottom row*, distances of side chains and the N5 carbon of folate are illustrated when MTHFR bound folate. Also, the folate-binding modes in the active sites are drawn in the *stick mode*. The carbon atom (C11) attached to the folate N5 is indicated as ∗. The distances between FAD N5 and folate C11 are 3.8 Å and 3.3 Å in tMTHFR•CHO-H_4_folate (*left*) and eMTHFR•CH_3_-H_4_folate (*right*), respectively. FAD, flavin adenine nucleotide; MTHFR, methylenetetrahydrofolate reductase; tMTHFR, Thermus thermophilus MTHFR.

The novel folate-binding mode affects the orientation of the folate relative to the FAD cofactor. As shown in Figure 4, *A* and *B*, conserved Asp and Gln residues (Asp109, Gln180 and Asp120, Gln183 in tMTHFR and eMTHFR respectively) anchor the folate molecule. However, the water arrangement near the interface of the folate pterin ring, the FAD isoalloxazine ring, and the L4 loop conformation are different in these two bacterial MTHFR structures (Fig. S7 and Table S1). The L4 loop conformation is important since it carries the invariant Asp residue and can therefore affect folate binding and activation (Fig. 1*B*). In eMTHFR, the L4 loop conformational diversity is dependent on ligand-binding and pH (14) (Fig. S7 and Table S1). When a water molecule is found between the Asp120 residue and the isoalloxazine ring of FAD, the L4 loop adopts an “open” conformation. When the L4 adopts a “closed” conformation, the water molecule is eliminated, and the Asp residue is proximate to FAD, forming a hydrogen-bonding network using Asp as a bridging residue between FAD and folate. When eMTHFR, for example, binds CH_3_-H_4_folate, the distance between Asp and FAD is 3.9 Å (Fig. 5*B*, from N1 of FAD to Asp120), and L4 is in the closed conformation (14). In tMTHFR•CHO-H_4_folate, where L4 adopts an open conformation, in addition to the water (Figs. 4*A* and S8, water 3) that bridges Asp109 and the FAD N1-C2=O, there is an additional water molecule (water 2) that is inserted between the folate and FAD (Fig. S8); the hydrogen-bonding network between FAD and folate is thus disrupted, and the additional water is hydrogen bonded to C4-O folate and the O atom of the FAD N1-C2=O moiety. As a result, the distance between the N1 of FAD and Asp109 increases to 5.3 Å. (Table S1); this removes the direct bridging Asp109 interaction between N2, N3 of folate, and N1 of FAD normally present when folate is bound (Fig. 4, bottom left).

The open L4 loop as well as the new/unique PABA pocket in tMTHFR•CHO-H_4_folate have a direct influence on the shape of the folate ligand in the active site. The angle of the PABA moiety to the plane of the pterin ring was estimated using the N10-C6-N3 dihedral (Fig. 4*C*). The pterin and PABA in CH_3_-H_4_folate are almost orthogonal, as indicated by the approximately right, 90∼93° angle they form, whereas those in CHO-H_4_folate are arranged in a more acute fashion/manner with an 84∼86° angle (Fig. 4, *C*-1 and *C*-2). As a result, the distances between N10 and C11 are 4.1 Å and 3.5 Å in CH_3_-H_4_folate and CHO-H_4_folate, respectively (Fig. 4, *C*-1 and *C*-2). Guided by the shorter N10-C11 distance in CHO-H_4_folate, a hypothetical conformation of the CH_2_-H_4_folate substrate was generated using the CHO-H_4_folate–binding mode as a mold to roughly approximate the position of the carbon attached to the N5 of folate in Figure 4*C*-3. In this model, the N10-C11 distance in CH_2_-H_4_folate is 1.5 Å, indicating that the novel binding mode of CHO-H_4_folate might represent a folate-binding mode more akin to that of CH_2_-H_4_folate or an intermediary state prior to its formation, given that its N11-C10 distance is in between that of CH_3_-H_4_folate and CH_2_-H_4_folate.

### Catalytic glutamate arrangement relative to folate & FAD

In the oxidative half reaction (Fig. 1*C*), CH_2_-H_4_folate, (Fig. 1*C*-5), after or concurrent to a protonation event, is converted to an iminium intermediate (Fig. 1*C*-6), followed by hydride transfer from FAD to the iminium intermediate to form CH_3_-H_4_folate (Fig. 1*C*-7). In the hydride transfer reaction (Fig. 1, *C*-6 and *C*-7), a hydride needs to be shuttled between the N5 FAD atom and the N5-C atom of folate. Therefore, the two ligands (FAD and folate) in the active site of MTHFR are in close proximity and oriented appropriately so as to enable electron transfer. The catalytically essential Glu residue (Glu18 in tMTHFR, Glu28 in eMTHFR), N5-C of folate, and N5 of FAD need to be in close proximity for hydride transfer in the folate-dependent reaction of MTHFR to occur. Indeed, the distance between N5-C of folate and N5 of FAD in the eMTHFR•CH_3_-H_4_folate structure is 3.3 Å (14), while that in the tMTHFR•CHO-H_4_folate structure is 3.8 Å in one monomer and 4.0 Å in the other (Fig. 5). The longer distance observed between folate N5-C and FAD N5 in the tMTHFR•CHO-H_4_folate structure indicates that the ligands and active site residues are not in an arrangement (as) conducive to hydride transfer. The catalytically essential Glu residue (23) (Glu18 and Glu28 in eMTHFR and eMTHFR, respectively) plays an important albeit not well understood role in the MTHFR catalytic cycle. In all available MTHFR•folate structures, this Glu (or Gln in eMTHFR•CH_3_-H_4_folate) is situated in between and in close proximity to both pterin and isoalloxazine rings (albeit in slightly different interacting positions). For example, the FAD N5 atom in tMTHFR is 3.8 Å apart from Glu18 Oε2, while in eMTHFR, it is 4.8 Å away from Gln28-Nε2 (the Glu residue is mutated to Gln) (Fig. 5). Although repositioning the Glu side chain against the isoalloxazine ring of FAD may relate to the catalytic process in the folate-dependent reaction, we propose that Glu’s reactivity is modulated through its interaction with an invariant Thr and through the formation of an active site quartet.

### Functional implications of an active site Ser-His-Glu-Thr quartet

In the tMTFHR•CHO-H_4_folate structure, the “open” state of the L4 loop reconfigures the hydrogen-bonding network between Asp109, folate, and FAD (Fig. 4*A*) slightly separating the folate pterin ring and FAD and disrupting an Asp109 bridging hydrogen network. Incidentally, another unique hydrogen-bonding network in the tMTFHR•CHO-H_4_folate structure arises from the rotation of Thr49 and its interaction with Glu18 to create a Ser-His-Glu-Thr quartet as shown in Figure 5 and Fig. S7. Indeed, the short distance of 2.6 Å between Thr49 Oγ to Glu18 Oε2 is indicative of a strong hydrogen bond (Fig. 5, lower-left panel). Through the engagement of Thr49 with Glu18, the active site triad previously consisting of Glu18, His270, and Ser16 is further extended. As a result, these residues in the tMTHFR•CHO-H_4_folate structure form a novel Ser-His-Glu-Thr quartet. Interestingly, the Ser-His-Glu triad is highly conserved in MTHFR, and its hydrogen-bond network further extends from the hydroxyl group of Ser16 to a surface water through an internal water molecule and the backbone peptide bond between Leu14 and Phe15 (Fig. S9). In contrast, in the folate-free tMTHFR structure, the distance between Thr and Glu is found to be 5.7 Å, suggesting that the triad expansion to include the interacting Thr49 and formation of a tetrad is triggered or is coupled to binding of a specific folate form. Indeed, the Thr coordination in the folate-free tMTHFR structure is very similar to that in the eMTHFR structure with or without CH_3_-H_4_folate (Fig. 5). It has to be noted that while the Thr residue exhibits a dynamic behavior, the positions of the Ser, His, and Glu residues in the triad remain largely unchanged.

A similar Thr rotamer found in the tMTHFR•CHO-H_4_folate structure that leads to the observed quartet can also be found in previously published substrate-free MTHFR structures, such as in the eMTHFR structure (12) (PDB 1B5T, at pH 6.0) and the holodimer tMTHFR structure at low resolution (PDB 3APY, at pH 8.0) (8). While the Thr side chain adopts a similar rotamer to the tMTHFR•CHO-H_4_folate structure, the distances between Thr and Glu residues in these models (1B5T and 3APY) are lengthy, at more than 3.5 Å. Thus, the Thr-Glu interaction in those structures is either weaker than that in the tMTHFR•CHO-H_4_folate complex or does not exist. Although the 1B5T and 3APY structures have been determined at near-natural pH, the quartet-containing tMTHFR•CHO-H_4_folate structure was solved at pH 4.5. However, a tMTHFR structure solved at pH 7.8 (Fig. S5) display similar features to the structure solved at pH 4.5/lower pH, namely the dimer mode 1 interface and the presence of the quartet. It is of note that the quartet formation is not found in the dimer 1 mode tMTHFR structure determined at pH 4.3∼4.5 without folate (8). The available MTHFR structures indicate that the Thr residue in MTHFR is free to rotate, adopting unique rotamers that give rise to different interaction networks in the active site and that multiple factors, predominantly ligand/cofactor-binding, affect the quartet formation (and dimer interface).

It has been proposed that the ionization state of Glu's carboxyl group plays a critical role in electrostatic stabilization of the 5-iminium intermediate. CHO-H_4_folate may be mimicking the reaction intermediate in the folate-dependent half reaction that results from (or forms after) CH_2_-H_4_folate ring opening (23). However, during the N5-N10 ring-opening reaction, the electronegative Glu near the methylene group may not be ideal due to potential undesirable side reaction (Fig. S10) that could occur if a water molecule is near the Glu residue. The engagement of Thr49 to Glu18 and the remodeling of the active site in their vicinity may allow for water exclusion near the iminium atoms and therefore prevent any byproduct formation. Moreover, the quartet formation allows for modulation of the Glu’s side chain electronegativity by forming an extended hydrogen bond network that extends from the vicinity of the bound ligands to bulk solvent (Figs. 5 and S9). Conceivably, MTHFR modulates the Glu residue reactivity *via* the quartet formation until hydride transfer is ready to occur.

## Discussion

### Folate tMTHFR inhibition is pH-dependent

The pH dependence of tMTHFR inhibition (Fig. 2, *B* and *C*) provides a possible clue as to how CHO-H_4_folate or CH_3_-H_4_folate can act as inhibitors of tMTHFR activity. We postulate that there is an ensemble of two folate-accessed conformations and that their relative population is affected by pH. Protein protonation can be responsible for the suggested conformational changes, although the nature of the amino acid residue(s) that is(are) protonated could not be identified in the present study.

Regarding the CH_3_-H_4_folate substrate inhibition that we observe, there are no available data to support that MTHFR activity is regulated by a second folate-binding site. We have found no crystallographic or conclusive biochemical data to suggest that folate binds anywhere else but in the active site. We therefore hypothesize that the observed folate-binding mode in our tMTHFR•CHO-H_4_folate structure is related to the observed inhibition by CHO-H_4_folate and by extension the substrate inhibition by CH_3_-H_4_folate. It is possible that at low pH, a large fraction of the enzyme is trapped as the inhibitory/nonproductive complex with CH_3_-H_4_folate, resulting in little to no turnover; higher pH would allow the inhibitory complex to disassemble or convert to the deprotonated tMTHFR form, relieving the observed substrate inhibition. Our model in where CH_3_-H_4_folate can access two pH-dependent folate-binding poses (or two protein ensembles) is consistent with the observed substrate inhibition based on the CH_3_-H_4_folate:menadione tMTHFR oxidoreductase assay. As previously mentioned, tMTHFR activity is much more sensitive to CHO-H_4_folate inhibition than human MTHFR. A possible explanation for the extreme sensitivity of tMTHFR to CHO-H_4_folate is its high isoelectric point (pI). While the calculated tMTHFR pI is 9.0 (29), the pIs for eMTHFR and the catalytic domain of human MTHFR are 6.0 and 5.5, respectively. At a pH lower than its pI, tMTHFR active site traps folate species such as CHO-H_4_folate and CH_3_-H_4_folate in a nonproductive form that it cannot escape, since it can either not activate or easily release them. It is worth noting that eMTHFR shows strong substrate inhibition for CH_2_-H_4_folate and NADH, but not CH_3_-H_4_folate. In addition, it has been reported that the apparent K_M_ for CH_2_-H_4_folate of pig MTHFR (calculated pI of 5.4) at pH 6.7 is 19 μM, which is substantially lower than that at pH 7.2, 88 μM (30). In either case, tMTHFR has shown to preferentially bind CH_2_-H_4_folate, with no associated substrate inhibition.

In the case of tMTHFR inhibition, protein protonation and CHO-H_4_folate (or CH_3_-H_4_folate) *per se* are not potent factors in isolation, but they become strong effectors when they cooperate/potentiate their respective effects. Despite binding in the active site, CHO-H_4_folate was found to be a noncompetitive inhibitor, causing a ligand-induced conformational change from dimer mode 2 to dimer mode 1. A similar effect was found previously, caused by FAD loading, where the change from a half-FAD occupancy (dimer mode 1) to an FAD-replete dimer would promote an identical shift in dimer interfaces (8). FAD, folate, and pH are proposed factors that can alter protein tMTHFR conformation and while the physiological significance of the heterodimer (dimer mode 1) formation is not clear, the dimer-interface–related conformational change could result in modulation of the catalytic function of the enzyme. It is possible that a half-sites’ reactivity model based on dimer mode 1 and the noncompetitive inhibition of CHO-H_4_folate may provide potential insights into a yet to be described allosteric regulatory mechanism in tMTHFR.

### Functional implications of the CHO-H_4_folate–binding mode

Despite its unique role as the bridge between the folate and methionine cycles, its importance in human health, and being a subject of study for decades, there is a dearth of information on the molecular MTHFR catalytic mechanism and how this MTHFR activity is regulated. In this paper, we describe the crystal structure of tMTHFR in complex with CHO-H_4_folate and the effects of CHO-H_4_folate on tMTHFR activity. These new structures showcase the structural flexibility of MTHFR, with a simple yet elegant solution to achieve regulatory control using a limited set of amino acid residues and rearrangements in the absence of any additional regulatory domains. Comparison of the tMTHFR•CHO-H_4_folate and eMTHFR•CH_3_-H_4_folate ternary complex structures reveals two distinct folate conformations as well as differences in the active site amino acid arrangements that accommodate them. The active site configurations for CH_2_-H_4_folate and CH_3_-H_4_folate binding have been expected to be different because the CH_2_-H_4_folate’s N5-N10 methylene bridge imposes torsional restriction affecting its shape (5). In the CHO-H_4_folate–binding mode, the altered Phe-rich hydrophobic PABA pocket and the repositioning of the critical Gln180 and Asp109 that anchor the folate pterin moiety affect the molecular shape of CHO-H_4_folate in the active site. Since the CHO-H_4_folate and CH_3_-H_4_folate pterin rings stack against the FAD *si*-face in a similar manner, the position of the PABA ring ultimately determines the dihedral angle between the PABA and pterin rings (Fig. 4*E*). Such PABA-ring–driven folate conformational change was previously proposed (14) and is consistent with our observations. Interestingly, the Phe31 side chain–promoting PABA ring repositioning is also observed in *E. coli* DHFR (18), suggesting the remodeling of the active site around the PABA ring may be a conserved strategy to control folate shape in folate-dependent enzymes.

In the tMTHFR•CHO-H_4_folate ternary structure, the active site arrangement is not favorable for hydride transfer between folate and FAD (Figs. 4 and 5), whereas the eMTHFR•CH_3_-H_4_folate structure could support it (Fig. 1*C*-7). Therefore, we posit that the active site conformation in the tMTHFR•CHO-H_4_folate structure represents a different folate functional state, either the substrate CH_2_-H_4_folate or the ring-opening folate intermediate-binding mode (Fig. 1, *C*-5 and *C*-6 respectively). In the mode represented by the CHO-H_4_folate–binding pose, an active site remodeling is manifested by the combination of the Phe20-containing PABA pocket arrangement, an open conformation of the L4 loop that affects pterin folate positioning, and the Ser-His-Glu-Thr quartet formation that allows for modulating the Glu reactivity. In sum, the unique folate-binding mode and structural active site remodeling in tMTHFR•CHO-H_4_folate structure compared to the eMTHFR•CH_3_-H_4_folate one are potentially indicative of how MTHFR binds and activates CH_2_-H_4_folate or the subsequent ring-opening iminium intermediate. Our hypothesis would be able to successfully rationalize the MTHFR biochemical properties of previous reports (8, 30) as well as the ones presented in our current study (Fig. 1*C*-7).

The ligand-induced conformational change upon CHO-H_4_folate and its noncompetitive inhibition in the NADH:menadione oxidoreductase assay were unexpected, as the change in dimer interface (dimer mode 1) is less extensive (than dimer mode 2); crystallographic evidence shows that CHO-H_4_folate is found only in the active site. Several other folate-binding enzymes, namely DHFR (18) and TYMS (31, 32), have shown that protein motions, in the form of conformational sampling in the active site (DHFR) or the existence of conformational ensembles (both) are crucial not only to catalysis but represent a route through which allostery can emerge. In the case of TYMS, the existence of a ligand-induced β-kink in the dimer interface directly couples the active sites between monomers and explains its observed half-sites reactivity (31), with an asymmetric dimer assembly structure elucidated (32). More studies are necessary to determine how noncompetitive inhibition in tMTHFR occurs and could prove invaluable in delineating the catalytic cycle of MTHFR itself. The modulation of enzyme activities through changes in a subunit interface might in turn be related to the allosteric regulation of human MTHFR, which is also a dimer.

### Summary

MTHFR provides a paradigm of how structurally dynamic changes/conformational sampling, induced by both ligand-binding and/or pH, can allow a single active site to accommodate chemically distinct species (in NADH and at least three different folates). MTHFR is able to perform chemically distinct transformations, including but not limited to carefully controlled redox chemistry and a difficult C-N bond/ring cleavage, acting as the sole source of a folate-based methyl donor. Using the same set of limited amino acids, MTHFR can perform the reaction *in reverse*, converting a methylated amine into an imidazoline ring (formal ring formation). The subtle but purposeful active site changes in MTHFR represent a rather impressive biological example and an adept demonstration of how intricate dynamic changes grant a single enzyme the ability to bind, stabilize, transform, and activate different substrates, using a delicate rearrangement of its active site to perform radically different chemistries. Despite increasing recognition of its important role in human health, the same features that enable MTHFR to bridge two metabolic cycles, namely its structural flexibility, have also hindered attempts to study its activity at the molecular level. Even so, the use of bacterial homologs and folate analogs have allowed one to trap MTHFR in action; simultaneously elegant and deceptively simple, our MTHFR work demonstrates Nature’s creativity as a “chemist”, providing us with lessons on how regulatory control can arise from local dynamic structural changes in the active site.

## Experimental procedures

### Chemicals

(*6RS*)-5-CHO-H_4_folate (folinic acid, calcium salt, Sigma-Fluka F7878-100mg), NADH (di-potassium salt, sigma N4505), and menadione (Sigma M5625) were purchased from Sigma. (*6S*)-5-CH_3_-H_4_folate (calcium salt) and FAD (di-sodium salt) were purchased from Eprova (Merck Eprova AG) and Usb corporation, respectively.

### Protein expression and protein purification

The expression vector, pET(tMR^wt^-H) (8) and pET(tMR^Glu18Gln^-H) were used to produce recombinant protein with an uncleavable hexa-Histidine tag on the C-terminus. *E. coli* BL21star(DE3) was transformed with the expression vector. Protein was induced in autoinduction media (33). Cells were propagated at 30 °C overnight and after collection by centrifugation, they were stored at −80 °C.

Protein purification was performed as previously reported (8). *E. coli* cells were suspended in 50 mM potassium phosphate buffer pH 7.4 (KPB) containing 0.2 M sodium chloride (NaCl) and then sonicated. The cell lysate was centrifuged, and the supernatant was pooled. After addition of free FAD, the supernatant was incubated at 70 °C for 15 min. Clear supernatant was obtained by centrifugation and filtration. The heat-treated, clear extract was loaded onto an Ni-affinity column (HiTrap Chelating, 5 ml, GE), which was equilibrated with 50 mM KPB, 0.2 M NaCl, and 50 mM imidazole. After washing the column with 50 mM KPB, 0.2 M NaCl, and 100 mM imidazole, the target protein was eluted with 50 mM KPB, 0.2 M NaCl, and 300 mM imidazole. The eluate was dialyzed against 0.1 M KPB, 0.1 M NaCl at 4 °C overnight. The dialyzate was concentrated and stored at 4 °C.

### Enzyme assay and UV-VIS spectroscopy

Enzyme assays were performed as previously described, using three distinct batches of purified enzyme to act as biological replicates (8). NADH:menadione oxidoreductase activity was measured using a Cary 100 Bio spectrophotometer (Agilent Technologies, Inc). The reaction mixture without menadione was prepared at room temperature. To start the reaction, menadione was added to the cuvette and then changes in absorption at 343 nm were recorded at room temperature. For routine assays, 100 μM NADH and 100 μl saturated menadione solution per ml of reaction mixture were used and data were fit according to Equation 1 (43):

(1) $$\frac{{v-v}_{f}}{v_{0}-v}=\frac{1}{1+\frac{\left[I\right]}{K_{I}}}$$

CH_3_-H_4_folate:menadione oxidoreductase activity was determined as follows (8): diluted tMTHFR in 50 mM KPB was preincubated at 50 °C and then menadione stock solution and enzyme were added. To start the reaction, (*6S*)-CH_3_-H_4_folate was added. The total volume of the reaction was 800 μl. The reaction was quenched by adding 200 μl of a 13 M formic acid and 5 M hydrochloric acid mixture, followed by heating at 90 °C for 10 min to convert the CH_2_-H_4_folate product to methenyltetrahydrofolate. After cooling, absorbance at 350 nm was measured. To calculate the concentration of methenyltetrahydrofolate, the molar absorption coefficient of 26.0 × 10^3^ was used. The following equation (Equation 2) for single substrate inhibition (34, 44) was used to fit the data in Figure 2*D*:

(2) $$\frac{v}{\left[E_{t}\right]}=\frac{\left[A\right]\left[B\right]k_{cat}}{K_{M,A}\left[B\right]+K_{M,B}\left[A\right]\left(1+\left[A\right]K_{I}\right)+\left[A\right]\left[B\right]}$$

### Preparation of tMTHFR complex with CHO-H_4_folate

Purified tMTHFR was treated with an excess amount of CHO-H_4_folate, then the mixture was passed through a gel filtration column (PD-10 (GE)), equilibrated with 50 mM KPB, pH 7.4 to remove free ligand. CHO-H_4_folate coeluted with tMTHFR from the gel filtration column, which was equilibrated with free CHO-H_4_folate buffer, indicating that tMTHFR with oxidized FAD forms a tight complex with CHO-H_4_folate. Comparison of the UV-VIS spectra, of the free CHO-H_4_folate *versus* protein-bound CHO-H_4_folate after gel filtration, at 290 nm (Fig. 2*A* inset, green line), allowed us to estimate that tMTHFR was approximately 75% replete. When crystals of the tMTHFR•CHO-H_4_folate complex were prepared, they grew under the presence of added excess CHO-H_4_folate in the crystallization solution. Electron density corresponding to CHO-H_4_folate was found only in the active site of tMTHFR, suggesting that the stoichiometry ratio of CHO-H_4_folate to a tMTHFR monomer is 1:1.

### Crystallization and data collection

Folate-free tMTHFR^wt^ crystals were grown as follows. FAD-replete tMTHFR (25 mg/ml) in 0.1 M KPB and 0.1 M NaCl was mixed in a 1:1 ratio with a reservoir solution (0.1 M Tris–HCl, pH 8.5, 3 M NaCl). Crystals were grown at 20 °C using the sitting-drop vapor diffusion method.

Folate-free tMTHFR^Glu18Gln^ crystals were grown as follows. As-purified tMTHFR^Glu18Gln^ was prepared in a final concentration of 13 mg/ml in 0.1 M KPB, 0.1 M NaCl, and 0.6 mM of CH_3_-H_4_folate and was then mixed in a 1:1 ratio with reservoir solution (0.1 M Hepes, pH 7.8, 1 M LiCl, and 20% PEG 6000). The crystals were grown at 20 °C using the hanging-drop vapor diffusion method. Crystals were transferred to a harvesting solution (the reservoir solution containing ≈20% glycerol) for 1 min and then flash-frozen in liquid nitrogen.

The tMTHFR•CHO-H_4_folate complex was obtained by cocrystallization. Specifically, FAD-replete tMTHFR at a concentration of 28 mg/ml and ∼77% CHO-H_4_folate replete was used. The protein solution was diluted to 20 mg/ml with 0.1 M KPB, 0.1 M NaCl, and 1.2 mM CHO-H_4_folate. Protein solution was then mixed in 1:1 ratio with a reservoir solution (0.1 M sodium acetate buffer, pH 4.5, 2.5 M NaCl, 0.2 M lithium sulfate, and 1.2% myo-inositol) at 4 °C. Crystals were transferred to a harvesting solution (the reservoir solution containing ≈20% glycerol) for 1 min and then flash-frozen in liquid nitrogen. Diffraction data for folate-free MTHFR^wt^ and the tMTHFR•CHO-H_4_folate complex were collected on the GM/CA-CAT 23-ID-D beamline, while diffraction data for folate-free MTHFR^Glu18Gln^ was collected on the LS-CAT 21-D-D beamline at the Advanced Photon Source, Argonne National Laboratory. Crystals were mounted under gaseous nitrogen at 100 K. X-ray data sets were recorded by a Pilatus 3-6m detector for the former and on an Eiger 9M detector for the latter, respectively.

### Processing, phase determination, model building, and refinement

Summary of processing and refinement statistics are shown in Table 1. All data sets were processed by XDS (35) save for the MTHFR^Glu18Gln^ data set, which was processed by DIALS (36). Initial phases were obtained with Phaser (37) for both MTHFR^wt^ data sets, while MOLREP (38) was used for MTHFR^Glu18Gln^. For a search model, tMTHFR subunit (PDB 3APY) (8) without FAD was used for the molecular replacement method. Refmac5 (39) in the CCP4i program suite was employed for the model refinement. Only the physiologically active (6*S*)-CHO-H_4_folate stereoisomer could be appropriately assigned to the electron density in each monomer, despite using the (6*RS*)-CHO-H_4_folate racemic mixture when preparing the complex with tMTHFR. Though tMTHFR^Glu18Gln^ was cocrystallized with CH_3_-H_4_folate and the presence of a small-molecule was found in the active site near FAD, partial occupancy/nondefinitive electron density did not allow for a clear ligand assignment. For manual model building, COOT (40) was used. The model quality was evaluated by MolProbity (41). All figures of protein structure were generated by PyMol (42).

**Table 1:** X-ray crystallography data collection and refinement statistics

	Folate-free	CHO-H_4_folate complex	Folate-free (pH7.8)
Data collection			
Beamline, APS	GMCA 23-IDD	GMCA 23-IDD	LS-CAT 21-D-D
Wavelength (Å)	1.0332	1.0332	1.1271
Temperature (K)	100	100	100
Resolution (Å)	47.03–2.09 (2.15–2.09)	46.09–1.45 (1.47–1.45)	89.06–1.90 (1.94–1.90)
Detector distance (mm)	400	250	180
Rotation range per image (°)	0.2	0.2	0.5
Total rotation range (°)	110	120	180
Exposure time per image (s)	0.5	0.2	
Space group	P212121	P212121	P212121
Cell dimensions (a/b/c Å)	60.5/91.3/130.3	45.1/89.6,/161.3	44.3/89.1/161.2
Cell dimensions (°)	α = β = γ = 90	α = β = γ = 90	α = β = γ = 90
Observed reflections	166,278 (10,273)	504,876 (21,120)	323,295 (18,087)
Unique reflections	42,742 (2924)	116,419 (5654)	49,988 (2948)
Rmeas (%)	13.7 (62.1)	9.8 (114.4)	9.1 (26.5)
Rmerge (%)	11.8 (50.2)	8.6 (97.9)	7.7 (22.3)
R.p.i.m. (%)	6.9 (31.6)	4.6 (58.1)	3.5 (10.4)
<I/σ>	7.6 (2.1)	10.7 (3.0)	12.7 (4.1)
CC(1/2)	0.993 (0.674)	0.995 (0.674)	0.996 (0.965)
Multiplicity	3.9 (3.5)	4.3 (3.7)	6.5 (6.1)
Completeness (%)	98.7 (88.4)	99.7 (99.3)	97.7 (91.3)
Overall B (Å^2^) (Wilson plot)	29.6	17.7	26.0
Refinement			
Resolution range	74.76–2.09	80.63–1.45	80.73–1.90
Number of reflections (work/test set)	40,563/2117	116,321/5915	47,388/2534
Rwork/Rfree (%)	18.4/22.4	13.1/17.8	20.9/25.0
No. of non-H atoms			
Protein	4534	9268	4489
Ligand	106	285	53
Water	62	445	231
B-factors (Å^2^)			
Protein	31.47	32.14	31.82
Ligand	30.92	28.09	33.55
Water	27.16	39.46	31.37
Rmsd deviations			
Bond lengths (Å)	0.009	0.011	0.010
Bond angles (°)	1.618	1.587	1.594
Ramachandran plot (Favored/allowed/outliers)	98.4/1.6/0.0	99.0/1.0/0.0	98.7/1.3/0.0
MolProbity Score	2.7 (99th percentile)	1.07 (99th percentile)	1.31 (99th percentile)
PDB	7TH5	7TH4	8EAC

Ligand: FAD; flavin adenine nucleotide, FFO; (*6S*)-5-formyltetrahydrofolate.

## Data availability

Structural data are deposited in the RCSB Protein Data Bank. PDB-ID-codes: 7TH4 (DOI: https://doi.org/10.2210/pdb7TH4/pdb), 7TH5 (DOI: https://doi.org/10.2210/pdb7TH5/pdb), 8EAC (DOI: https://doi.org/10.2210/pdb8eac/pdb)

## Supporting information

This article contains supporting information (45).

## Conflict of interest

The authors declare that they have no conflicts of interest with the contents of this article.
