# Supplementary material for: 5-Formyltetrahydrofolate promotes conformational remodeling in a methylenetetrahydrofolate reductase active site and inhibits its activity
Source: J Biol Chem. 2022 Dec 31;299(2):102855. doi: 10.1016/j.jbc.2022.102855 (PMC9900621; doi:10.1016/j.jbc.2022.102855)
Supplement: Supplemental Tables S1, S2, Figures S1–S10 [file mmc1.docx]

**5-formyltetrahydrofolate promotes conformational remodeling in a Methylenetetrahydrofolate Reductase active site and inhibits its activity**

**Kazuhiro Yamada^1,2*^, Johnny Mendoza^1^, Markos Koutmos^1,2,3,*^**

^1^ Department of Chemistry, University of Michigan, Ann Arbor, MI, United States

^2^ Program in Biophysics, University of Michigan, Ann Arbor, MI, United States

^3^ Program in Chemical Biology, University of Michigan, Ann Arbor, MI, United States

**^*^** to whom correspondence should be addressed: Markos Koutmos, [mkoutmos@umich.edu](mailto:mkoutmos@umich.edu); Kazuhiro Yamada, yamadak@umich.edu.

**Index**

Figures S1-S10

Table S1 and S2


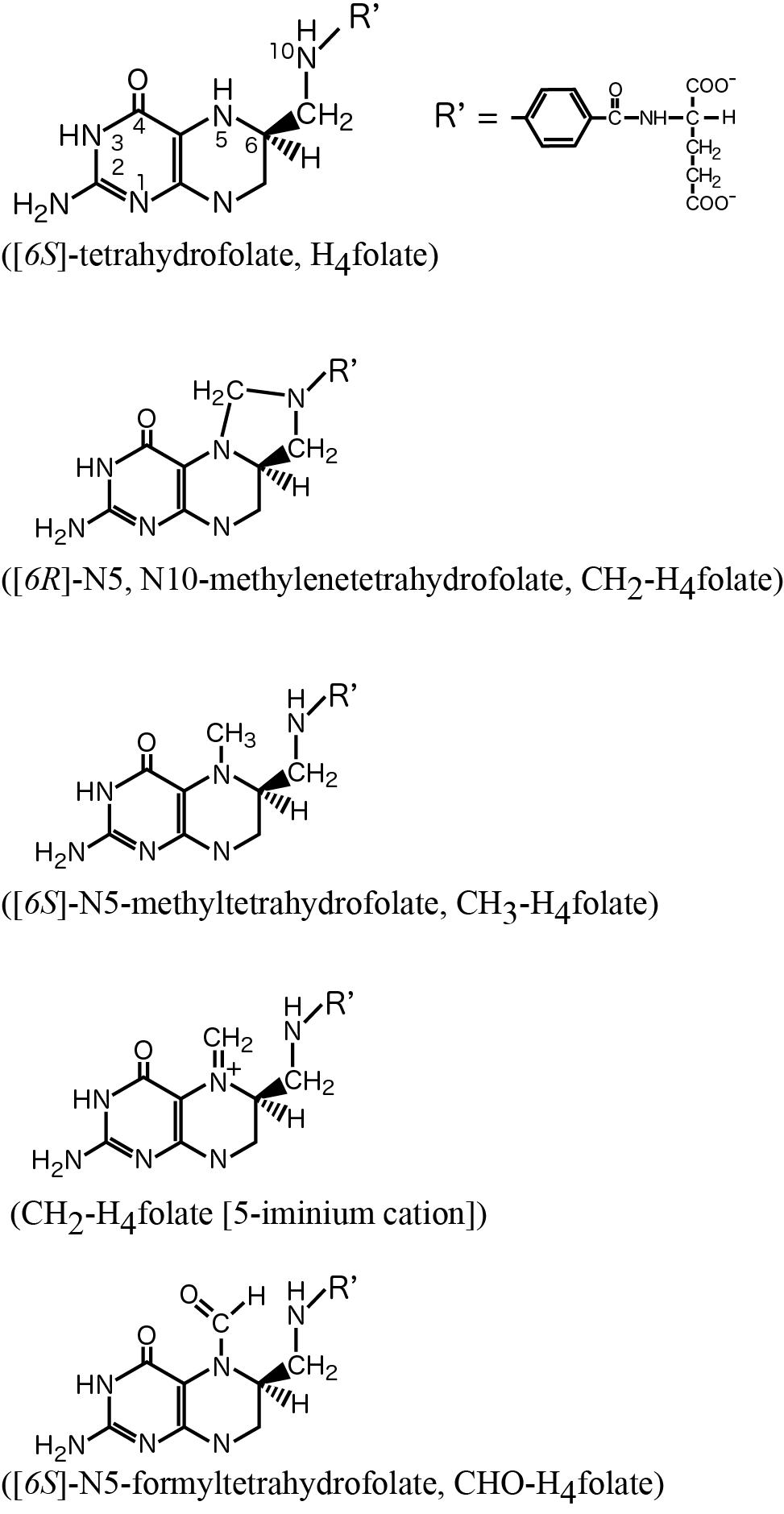


**Figure S1. Folate structures.** Chemical drawing of tetrahydrofolate, methylenetetrahydrofolate, methyltetrahydrofolate, methylenetetrahydrofolate (5-iminium cation) and formyltetrahydrofolate are shown.


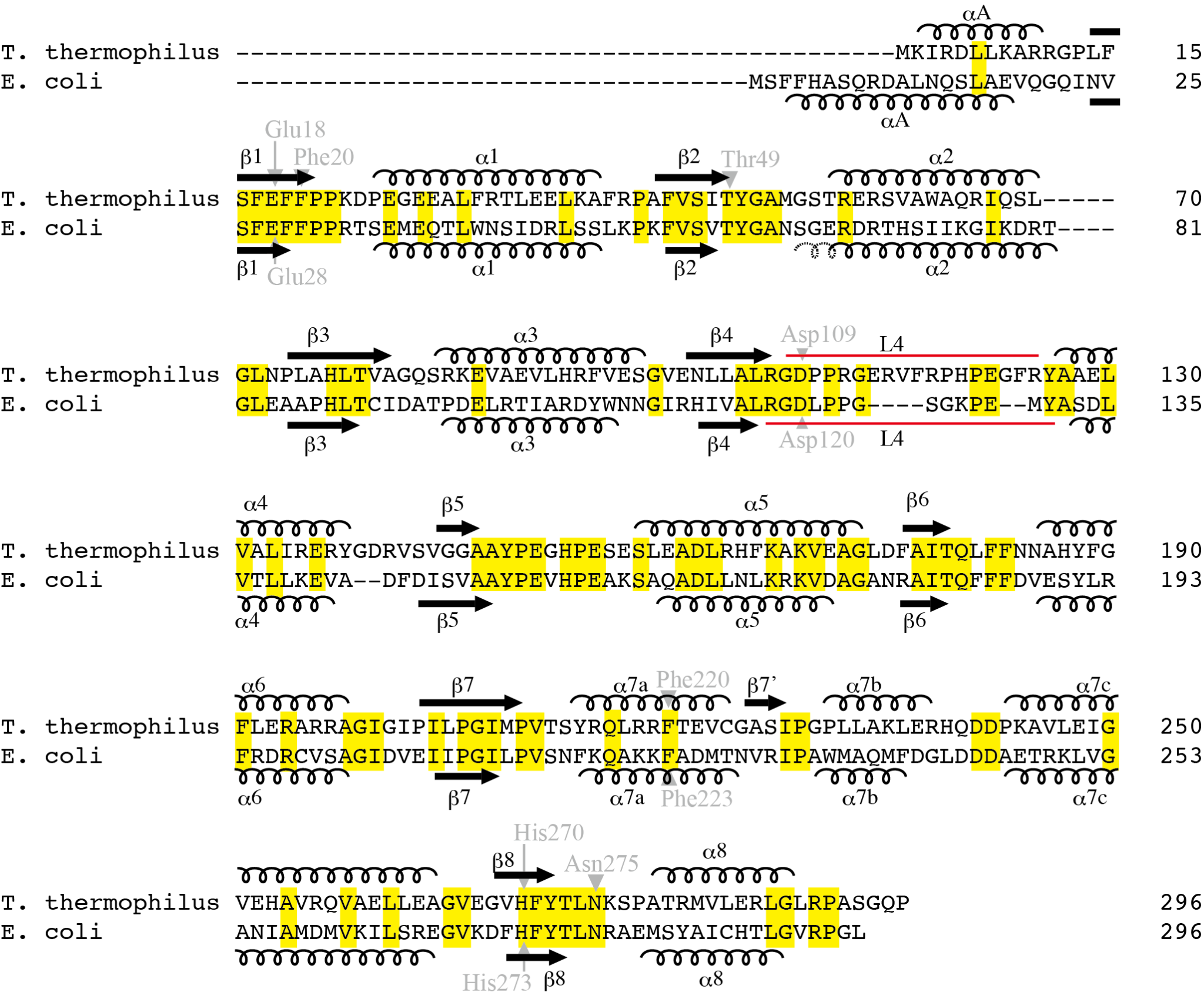


**Figure S2**. **Sequence Alignment.** Amino acid alignment of MTHFR from *T.thermophilus* (T. thermophilus. NCBI accession number: WP_011174022) and *E.coli* (E.coli. NCBI: NP_418376). Loop L4 is highlighted with the red line. Conserved amino acid residues are highlighted in yellow.


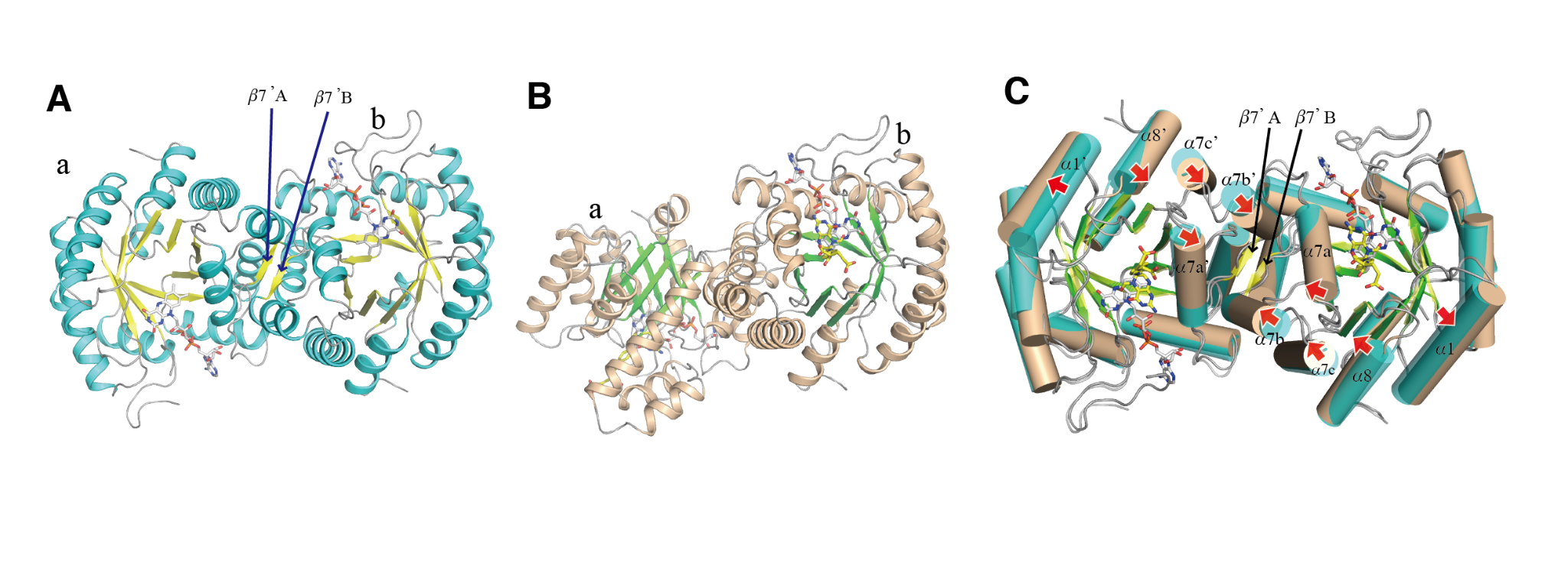


**Figure S3**. **The two different dimerization modes for tMTHFR** Comparison of tMTHFR structures with and without CHO-H_4_folate. Structures of **A** folate-free tMTHFR (full FAD occupancy dimer, dimer mode 2) and **B** tMTHFR•CHO-H_4_folate (dimer mode 1) displayed as cartoons. Helices and sheets are colored by cyan and yellow in **A** and light-brown and green in **B**. FAD (gray) and CHO-H_4_folate (yellow) ligands are shown as sticks. The “b” monomers in **A** and **B** are displayed in the same orientation. **C**. A theoretical dimer of the tMTHFR•CHO-H_4_folate structure in dimer mode 2 was generated using the folate-free tMTHFR structure in dimer mode 1 depicted in **A** as the template. The individual monomers (light-brown) in the tMTHFR•CHO-H_4_folate (Panel B-a and -b) were superimposed to the corresponding monomers of the folate-free structure (Panel A-a and -b) to model the tMTHFR•CHO-H_4_folate in the dimer mode 1 using the folate free structure as a guide. Helices in C are displayed as cylinders. Red arrows represent conformational changes of helices within each monomer that take place upon binding of CHO-H_4_folate (from cyan to light brown).


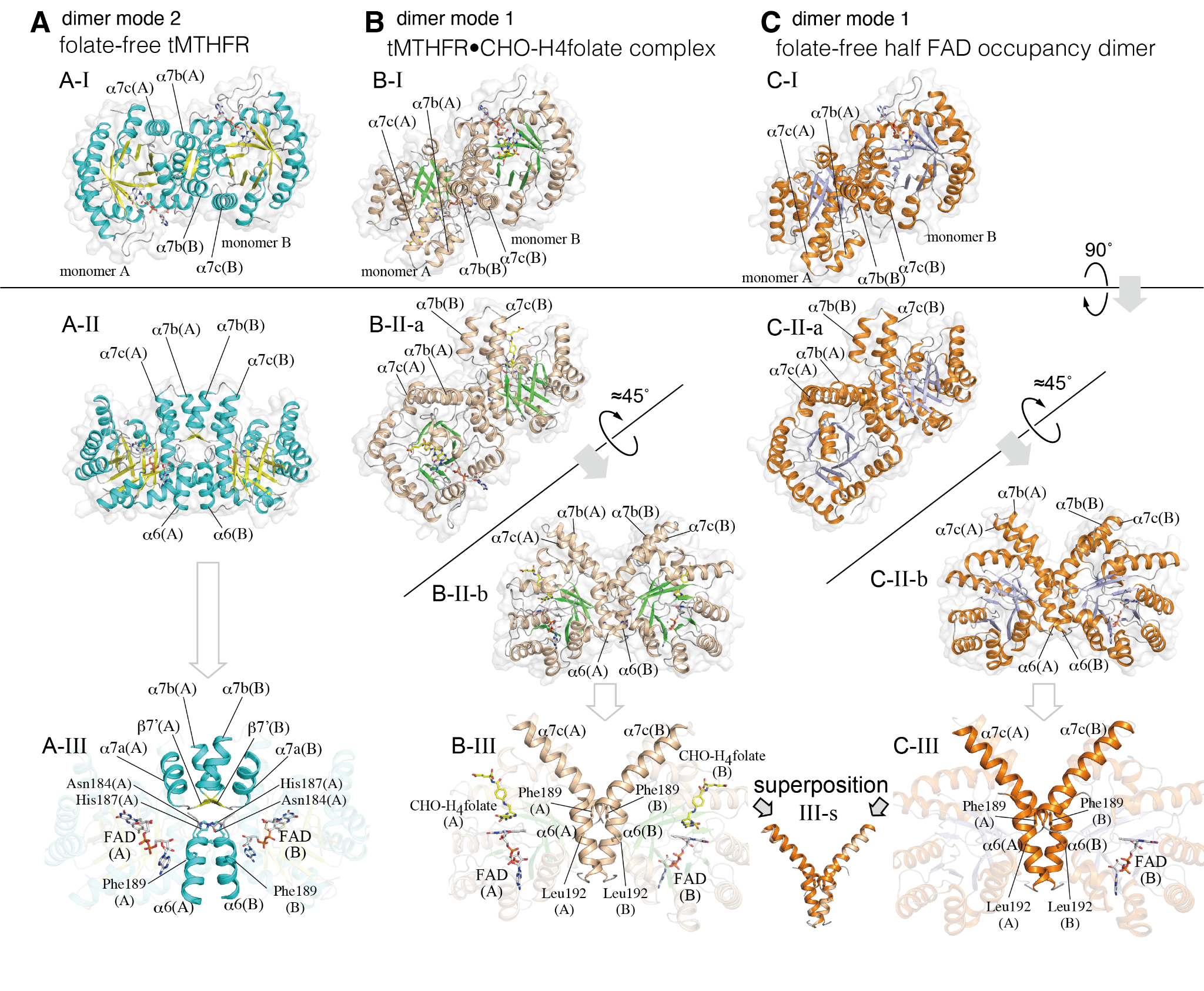


**Figure S4. Detailed comparison of tMTHFR dimer interfaces**. Structures of folate-free tMTHFR (A-I) and the tMTHFR•CHO-H_4_folate complex (B-I) that exhibit dimer mode 1 are shown in the same orientation as Figures 3S-A and B, but with the additional display of a transparent surface mode. The folate-free half FAD occupancy tMTHFR dimer in dimer mode 2 is illustrated in Panel C. The monomer B in Panel C-1 is drawn in the same orientation as monomers B in Panels A-I and B-I. The monomer A in C-I is the apo-monomer that lacks the FAD cofactor. In Panel C, helices and sheets are colored orange and pale blue respectively. Panels A-II, B-II-a/b, and C-II-a/b display different views of the dimer interfaces. Details of the different dimer interfaces are highlighted in Panels A-III, B-III, and C-III. In Panel III-s the superposition of only the B-III and C-III dimer interfaces in solid colors displays their perfect alignment and identical features.


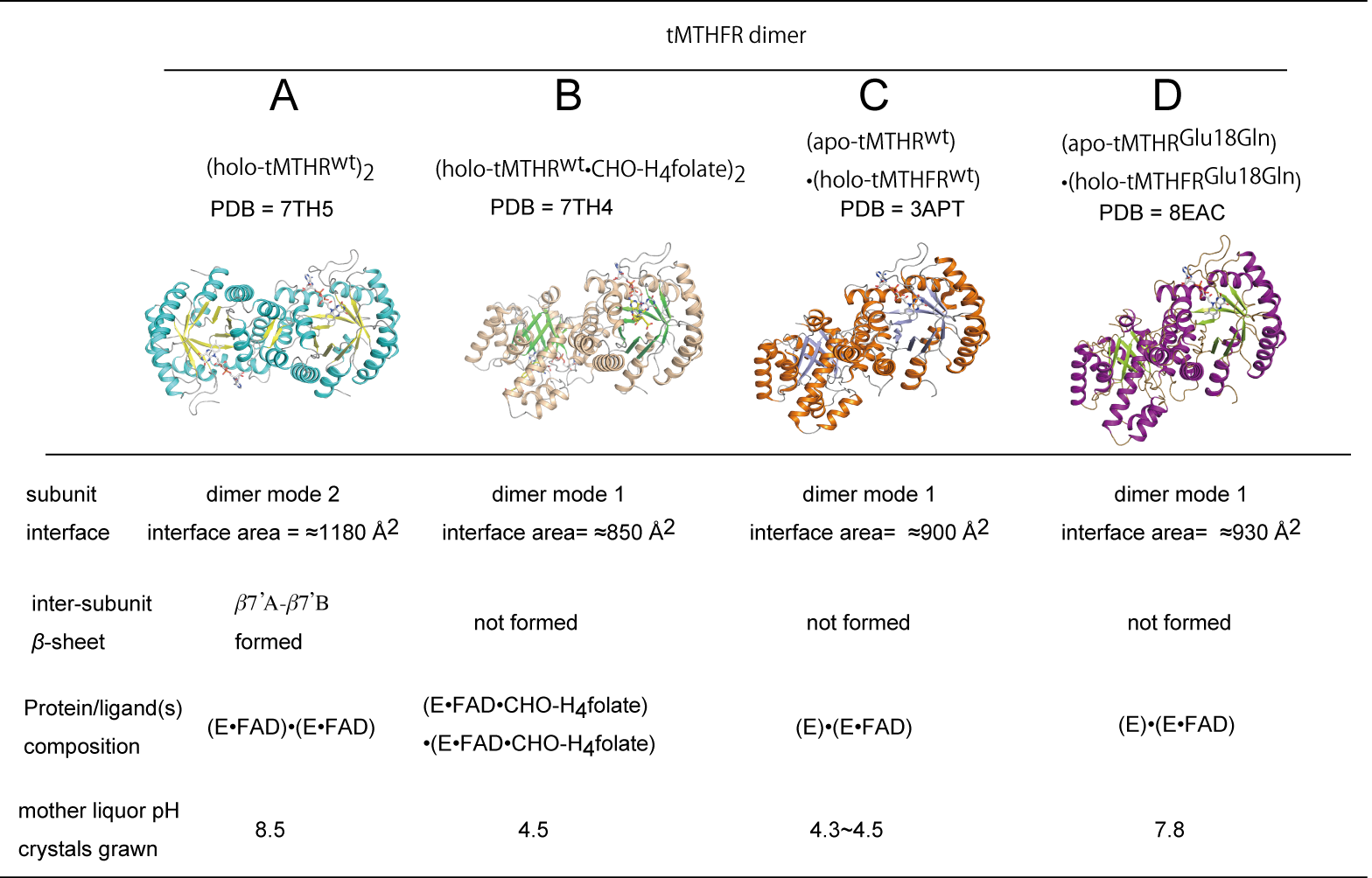


**Figure S5**. **Dimerization modes of tMTHFR structures at varying pH.** - The dimerization modes of tMTHFR are summarized. When both subunits bind FAD but without folate, tMTHFR forms a dimer in dimer mode 2 (Column A, PDB 7TH5). In dimer mode 2, the interface area is ≈1180Å^2^, and there is *β*-sheet formation in the interface. Upon CHO-H_4_folate binding (Column B, PDB 7TH4), the dimerization mode is altered to dimer mode 1. The dimer mode 1 is the same mode that we have previously reported the folate-free tMTHFR structure consisting of apo- and holo-subunits (Column C, PDB 3APT). In dimer mode 1, the interface area is ≈900 Å^2^, and no inter-subunit β-sheet formation exists. The tMTHFR dimer (dimer of the apo-subunit and the holo-subunit) employs the dimer mode 1 (Column D, PDB 8EAC). The interface area was calculated by PISA (E. Krissinel and K. Henrick (2007). Inference of macromolecular assemblies from crystalline state. *J. Mol. Biol*. **372**, 774-797).


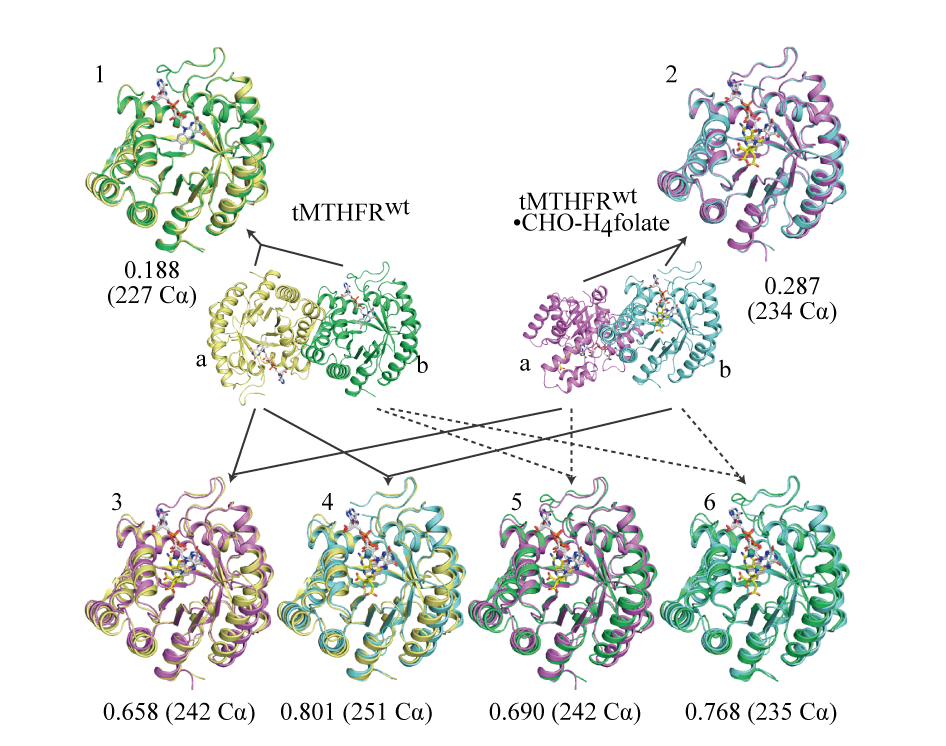


**Figure S6**. **Effect of CHO-H_4_folate binding on monomer structure.** Paired-comparison of tMTHFR monomers with or without CHO-H_4_folate. Monomers in the folate-free tMTHFR holo-dimer are colored yellow and green for monomer-a and -b, respectively. Monomer-a and -b in the of tMTHFR•CHO-H_4_folate complex are in magenta and cyan. Arrows indicate the combination of monomers for the paired superposition. Numbers are r.m.s.d. values, and numbers of Cα used for calculation are in parenthesis.


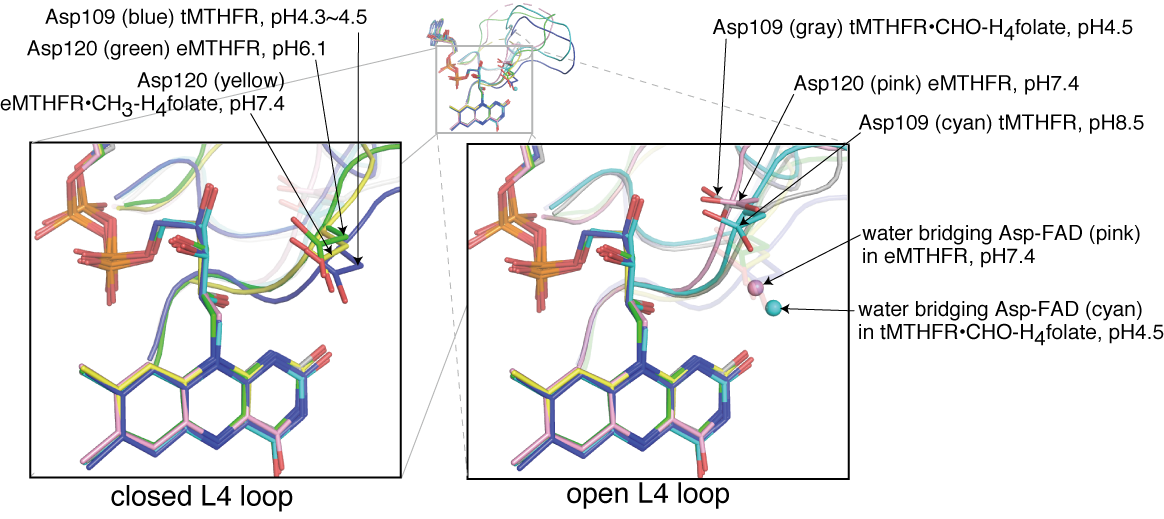


**Figure S7. Variability in L4 loop conformations.** The L4 loop conformations based on all available eMTHFR and tMTHFR structures, with or without folate and determined in varied pHs are shown. In this tMTHFR superposition focusing on the loop L4, the FAD ligand was used as a reference to align the monomer structures. Color schemes are the same as in Table 1S.


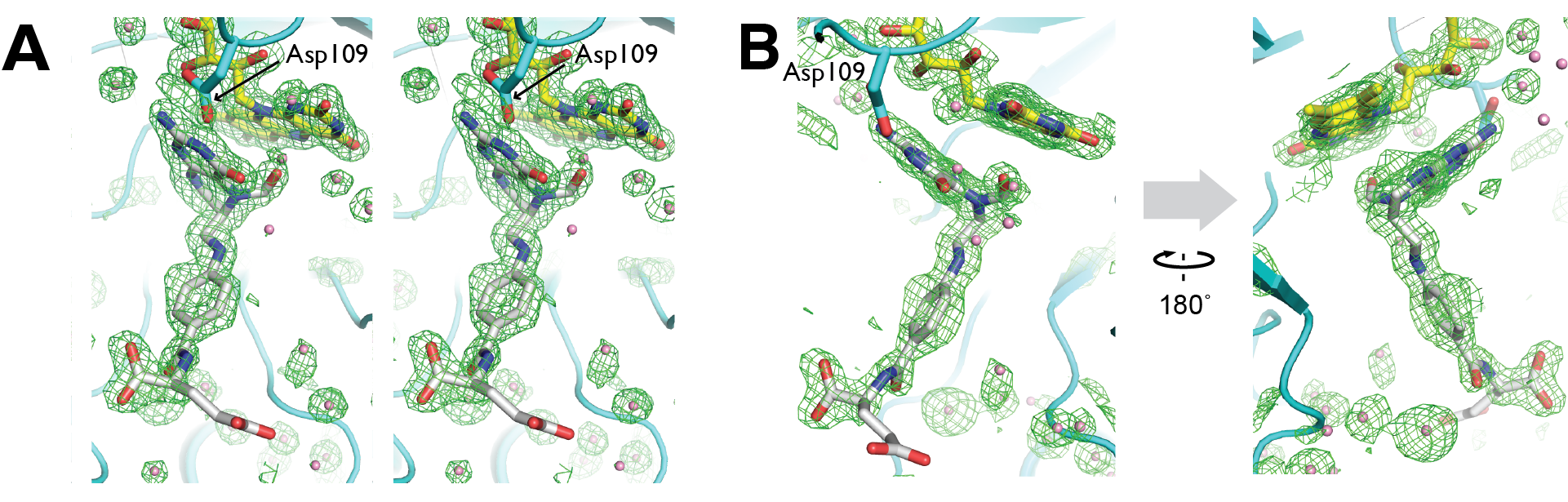


**Figure S8. Polder map of CHO-H_4_folate in the active site**. FAD and CHO-H_4_folate found in the active site are drawn in stick mode along with the electron density from an omit map (|Fo|-|Fc| map, 2.0 σ) A= A stereo view showing FAD and CHO-H_4_folate in the active site. The FAD cofactor and CHO-H_4_folate are shown in stick mode (yellow and gray, respectively). Water molecules are drawn in pink. Asp109 and protein backbone structure are colored in cyan. B= 2D projection image of the same omit map in the panel A. The color scheme is the same as in the panel A.


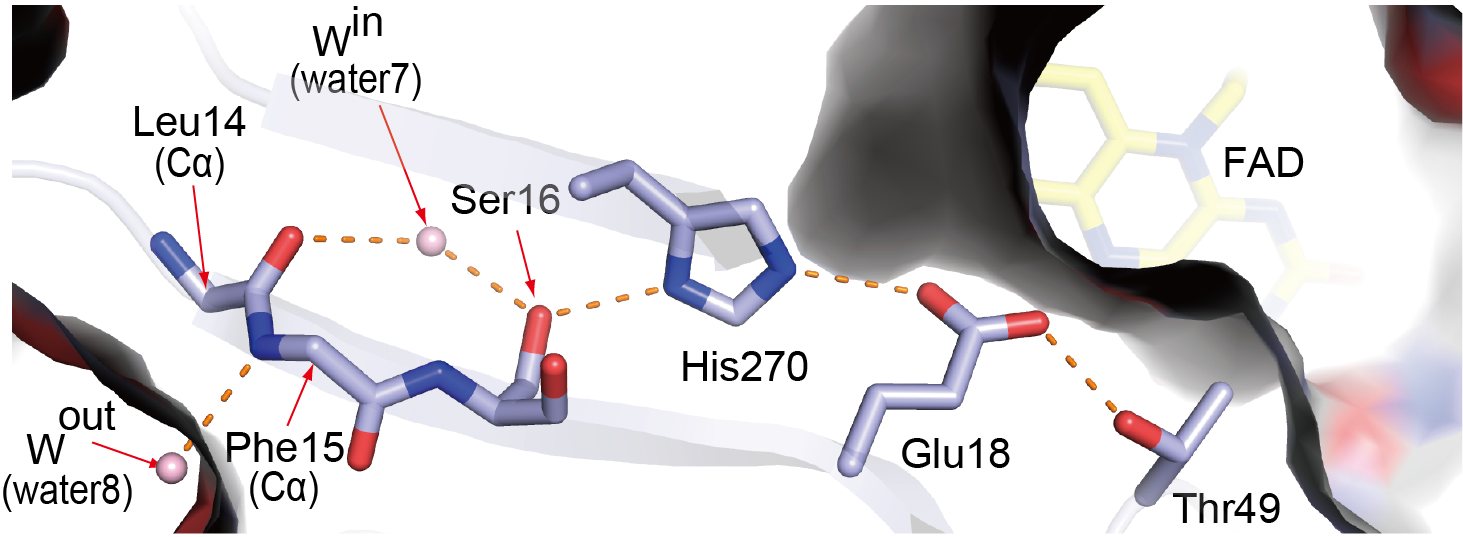


**Figure S9.** **Close-up view of the active site quartet**. A hydrogen bond network from Thr49 to the surface water. The hydrogen bond in the Thr-Glu-His-Ser quartet can be extended to the surface water (W^out^, water 8). The hydroxy group of Ser interacts with the internal water (W^in^, water 7). Then, the hydrogen bond network reaches water8 via the backbone peptide bound of Leu14 and Phe15.


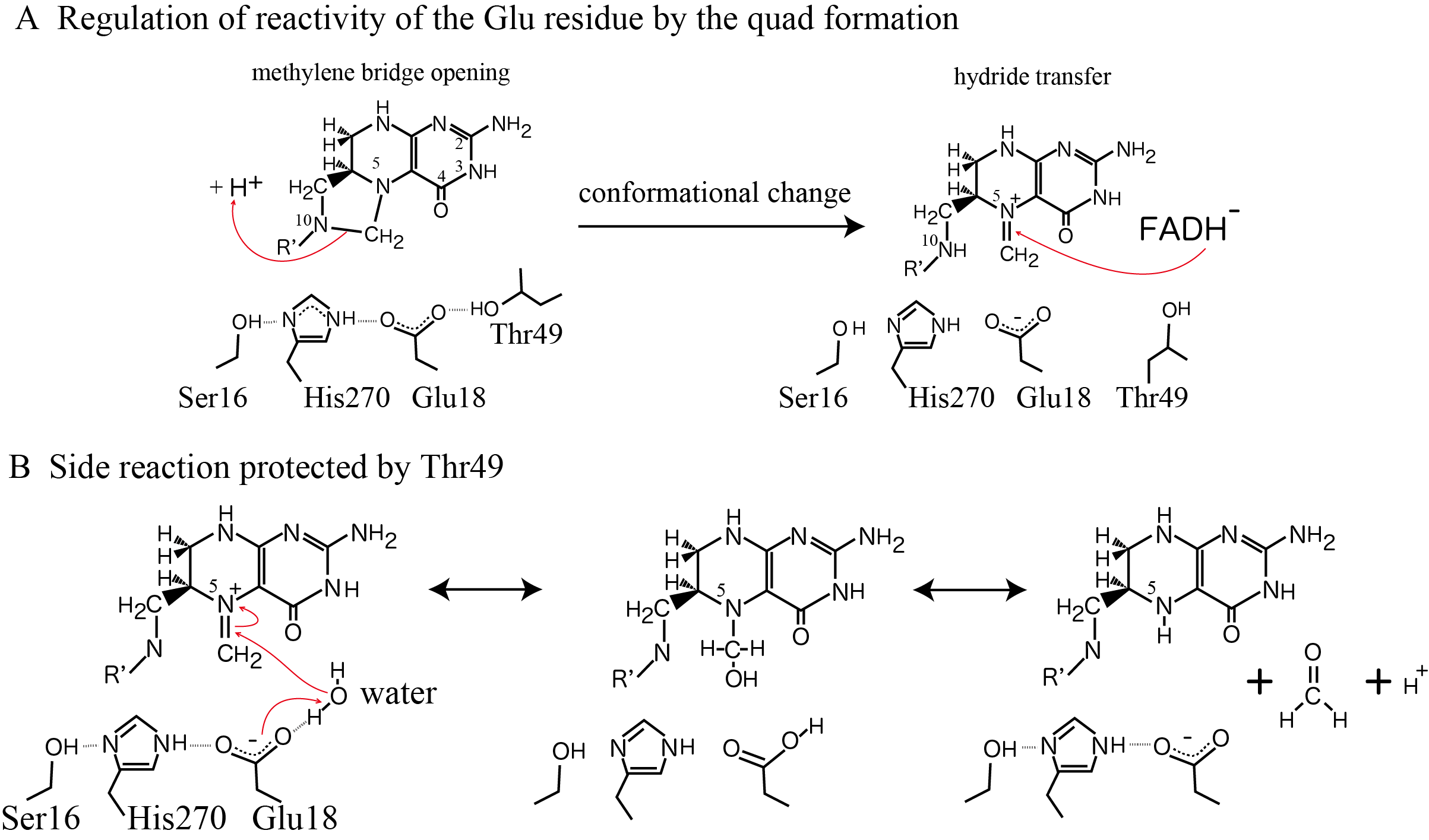


**Figure S10 Putative functional roles of the active site quartet.** A. During the N5-N10 ring opening, the quartet forms. After forming the 5-iminium intermediate, a conformational change is induced, and N5-C of folate gets closer to the Glu residue and FAD to process the reaction. The quartet could be disassembled due to the conformational change. B. Scheme for the possible side reaction that the Thr residue would protect. After ring opening, CH_2_-H_4_folate in the 5-iminium form converts to 5-hydroxymethyl-H_4_folate, which eventually decomposes into H_4_folate and formaldehyde.


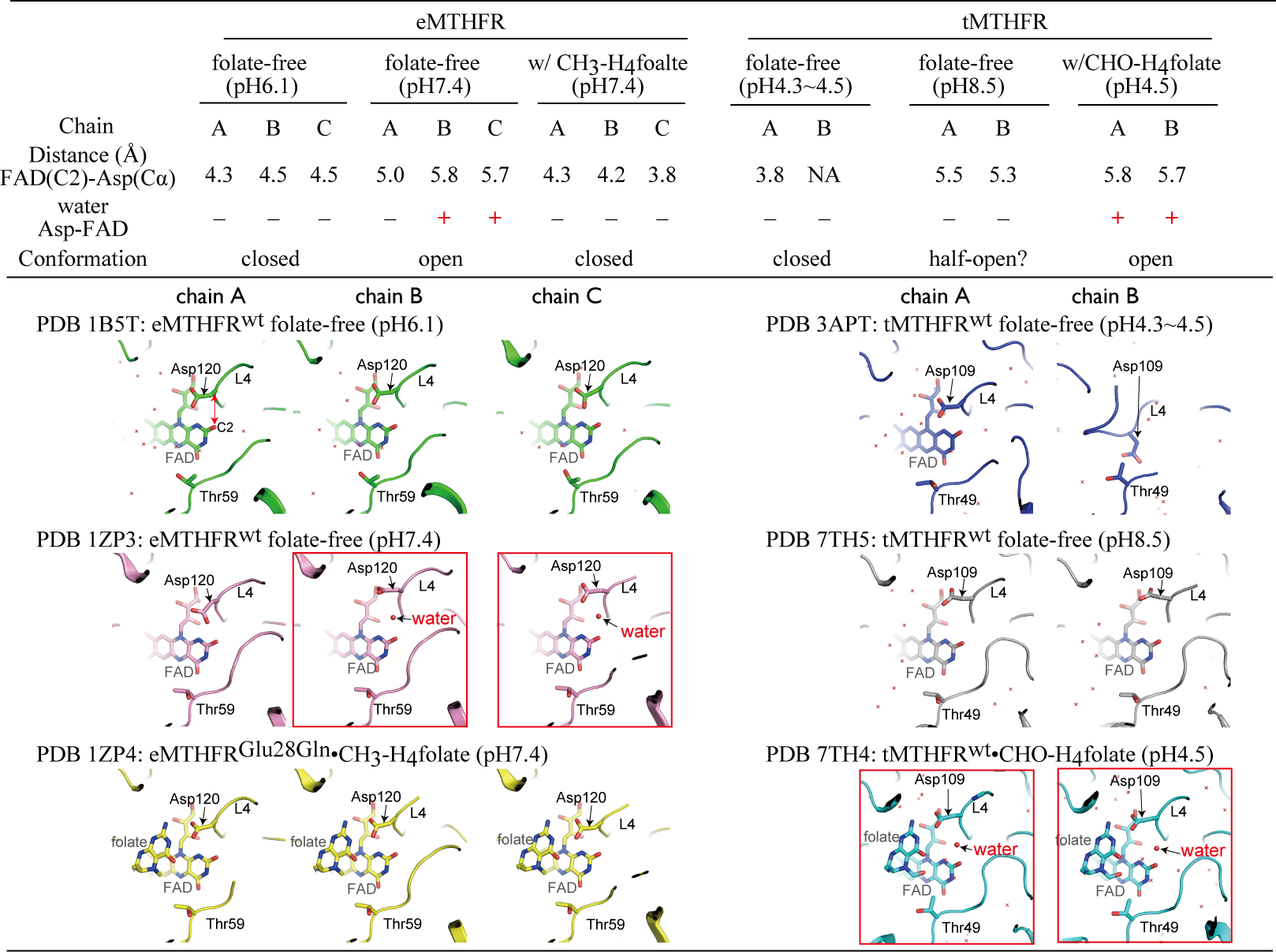


**Table S1**. **Comparative summary of L4 conformations.** **Top.** Selected distances between FAD and Asp are listed for all available bacterial MTHFR structures. The L4 conformation is denoted as open and closed. Waters in the Asp/FAD vicinity are indicated with red + symbols when present. **Bottom**. Close-up view of the Asp/FAD pair with surrounding waters represented as red spheres. The red arrow denotes the listed in the top part of the table distances between the FAD O2 and the Asp Cα atoms. Red squares surround the open conformations in where water molecules are found near the displayed Asp residue.

Table S2. Effect of pH on the CH_3_-H_4_folate:menadione oxidoreductase assay.
